# Root iTRAQ protein profile analysis of two *Citrus* species differing in aluminum-tolerance in response to long-term aluminum-toxicity

**DOI:** 10.1186/s12864-015-2133-9

**Published:** 2015-11-16

**Authors:** Huan-Xin Jiang, Lin-Tong Yang, Yi-Ping Qi, Yi-Bin Lu, Zeng-Rong Huang, Li-Song Chen

**Affiliations:** Institute of Plant Nutritional Physiology and Molecular Biology, Fujian Agriculture and Forestry University, Fuzhou, 350002 China; College of Life Science, Fujian Agriculture and Forestry University, Fuzhou, 350002 China; College of Resource and Environmental Science, Fujian Agriculture and Forestry University, Fuzhou, 350002 China; Institute of Materia Medica, Fujian Academy of Medical Sciences, Fuzhou, 350001 China; The Higher Educational Key Laboratory of Fujian Province for Soil Ecosystem Health and Regulation, Fujian Agriculture and Forestry University, Fuzhou, 350002 China; Fujian Key Laboratory for Plant Molecular and Cell Biology, Fujian Agriculture and Forestry University, Fuzhou, 350002 China

**Keywords:** Aluminum-toxicity, *Citrus*, iTRAQ, Proteomics, Root, Sulfur metabolism

## Abstract

**Background:**

Limited information is available on aluminum (Al)-toxicity-responsive proteins in woody plant roots. Seedlings of ‘Xuegan’ (*Citrus sinensis*) and ‘Sour pummelo’ (*Citrus grandis*) were treated for 18 weeks with nutrient solution containing 0 (control) or 1.2 mM AlCl_3_ · 6H_2_O (+Al). Thereafter, we investigated *Citrus* root protein profiles using isobaric tags for relative and absolute quantification (iTRAQ). The aims of this work were to determine the molecular mechanisms of plants to deal with Al-toxicity and to identify differentially expressed proteins involved in Al-tolerance.

**Results:**

*C. sinensis* was more tolerant to Al-toxicity than *C. grandis*. We isolated 347 differentially expressed proteins from + Al *Citrus* roots. Among these proteins, 202 (96) proteins only presented in *C. sinensis* (*C. grandis*), and 49 proteins were shared by the two species. Of the 49 overlapping proteins, 45 proteins were regulated in the same direction upon Al exposure in the both species. These proteins were classified into following categories: sulfur metabolism, stress and defense response, carbohydrate and energy metabolism, nucleic acid metabolism, protein metabolism, cell transport, biological regulation and signal transduction, cell wall and cytoskeleton metabolism, and jasmonic acid (JA) biosynthesis. The higher Al-tolerance of *C. sinensis* may be related to several factors, including: (*a*) activation of sulfur metabolism; (*b*) greatly improving the total ability of antioxidation and detoxification; (*c*) up-regulation of carbohydrate and energy metabolism; (*d*) enhancing cell transport; (*e*) decreased (increased) abundances of proteins involved in protein synthesis (proteiolysis); (*f*) keeping a better balance between protein phosphorylation and dephosphorylation; and (*g*) increasing JA biosynthesis.

**Conclusions:**

Our results demonstrated that metabolic flexibility was more remarkable in *C. sinenis* than in *C. grandis* roots, thus improving the Al-tolerance of *C. sinensis*. This provided the most integrated view of the adaptive responses occurring in Al-toxicity roots.

**Electronic supplementary material:**

The online version of this article (doi:10.1186/s12864-015-2133-9) contains supplementary material, which is available to authorized users.

## Background

In many acidic soils through the tropics and subtropics, aluminum (Al)-toxicity is a major factor limiting crop productivity. Approximately 30 % of the world’s total land area is acidic [1]. Furthermore, the acidity of the soils is increasing due to the environmental problems including some farming practices and acid rain [2]. When soil pH is less than 5.0, toxic forms of Al (mainly Al^3+^) are solubilized into the soil solution and accumulate to high concentration that rapidly inhibits plant root growth by damaging the roots functionally and structurally, subsequently decreasing nutrient and water uptake, eventually resulting in poor crop growth and productivity [3, 4]. A traditional strategy is to raise soil pH by application of lime, thus alleviating Al-toxicity; however, the approach is both costly and ecologically unsound from the long-term point of view. A more efficient strategy is to breed Al-tolerant crop cultivars. Fortunately, plants display wide variation in their ability to deal with Al-toxicity. Variation in Al-tolerance makes it possible to breed tolerant cultivars. The success of breeding programs relies on an understanding of the physiological, biochemical and molecular mechanisms that plants tolerate Al-toxicity. Since biological processes are ultimately controlled by proteins, identification and characterization of Al-tolerant proteins will not only increase our understanding of the molecular mechanisms on plant Al-tolerance, but also will provide new information that researchers will use to screen and breed crop cultivars suited for acidic soils with higher active Al.

During the long-term evolutionary process, higher plants have evolved two main strategies (i.e., external detoxification mechanisms and internal detoxification mechanisms) that enable them to tolerate high level of soil active Al [2, 3]. Although several mechanisms for the external detoxification (i.e., formation of non-toxic Al chelates with Al ligands released by roots, alkalinization of the rhizosphere, modified cell wall and redistribution of Al) have been suggested, the Al-induced secretion of organic acid anions such as citrate, malate and oxalate from roots is the most-studied mechanism of Al-tolerance in higher plants [2, 5]. Genes involved in Al-induced root secretion of citrate and malate have been isolated from wheat (*ALMT1*), sorghum (*SbMATE*) and barley (*HvMATE*). Transgenic plants over-expressing these genes displayed increased Al-activated root secretion of malate and/or citrate and enhanced Al-tolerance [2, 5, 6]. Internal detoxification is mainly reached by both complexation and sequestration of Al [6].

Gene expression networks revealed by transcriptomics offer us the opportunity to understand the molecular mechanisms of plant Al-toxicity and -tolerance. Although Al-induced changes in gene expressions have been examined in some detail [7–9], limited data are available on Al-toxicity-responsive proteins in higher plants. Alteration of plant proteins is an inevitable process to deal with environmental stresses including Al-toxicity [4, 10, 11]. Proteomics is a very powerful tool for analyzing the functions of the plant proteins. Abundance of a protein is not only regulated at transcriptional, but also at translational and post-translational levels. Therefore, proteomic analysis may provide more accurate and comprehensive data than what genomic studies can provide. Recently, there were several studies reporting Al-induced changes in plant proteins using two-dimensional gel electrophoresis (2-DE) or isobaric tags for relative and absolute quantification (iTRAQ) technique. Study with Al-tolerant rice (*Oryza sativa*) cultivar showed that root abundances of copper/zinc superoxide dismutase (Cu/Zn SOD), S-adenosylmethionine synthetase, cysteine (Cys) synthase, 1-aminocydopropane-1-carboxylate oxidase (ACC oxidase), glutathione S-transferase (GST) increased in response to Al-toxicity, and that Cys synthase might play a key role in Al-tolerance [4]. In Al-tolerant soybean cultivar roots, Al induced the production of heat shock proteins (HSPs), GST, chalcone-related synthetase, GTP-binding protein, and ATP Binding Cassette (ABC) transporter ATP-binding protein [12]. Duressa et al. [13] observed that Al resulted in a distinct protein profile changes in Al-tolerant and Al-sensitive soybean genotypes. In tomato roots, dehydroascorbate reductase (DHAR), glutathione reductase (GR), catalase (CAT), mitochondrial aldehyde dehydrogenase (ALDH), catechol oxidase, quinone reductase, and lactoylglutathione lyase involved in antioxidation and detoxification were induced by Al [14]. Dai et al. [15] isolated 35 proteins associated with Al-tolerance in Al-tolerant wild barley XZ16. There were 16 proteins, which were up-regulated in XZ16 roots, but down-regulated or unaltered in both Al-tolerant barley cultivar Dayton and Al-sensitive wild barley XZ61. Oh et al. [16] identified 19 up-regulated protein spots such as S-adenosylmethionine, oxalate oxidase (OXO), malate dehydrogenase (MDH), Cys synthase, ascorbate peroxidase (APX) and 28 down-regulated protein spots such as HSP 70, O-methytransferase 4, enolase and amylogenin in + Al wheat roots. Wang et al. [11] identified 106 Al-toxicity-responsive proteins from Al-sensitive and -tolerant rice roots. They observed that glycolysis/gluconeogenesis was the most significantly up-regulated biochemical process in Al-toxic roots. To our knowledge, data available on the effects of Al-toxicity on root proteomics of woody plants are very limited.

*Citrus* spp. are cultivated mainly in acidic and strong acidic soils such as red soil, yellow soil or lateritic red soil. In 2011, Li et al. [17] investigated the pH of 319 ‘Guanximiyou’ pummelo (*Citrus grandis*) orchard soils from Pinghe county, Zhangzhou, China. Up to 90 % of soils had a lower pH than 5.0. Low pH and high Al are the factors contributing to poor *Citrus* growth and shortened lifespan of trees [18]. In this study, we compared Al-induced quantitative and qualitative changes in proteomes that occurred in roots of Al-tolerant *Citrus sinensis* and Al-sensitive *C. grandis* seedlings [19] using iTRAQ technique in order to identify differentially expressed proteins involved in Al-tolerance and to understasnd the molecular mechanisms of plants to deal with Al-toxicity.

## Results

### Plant growth and Al concentration in roots

**Fig. 1:**
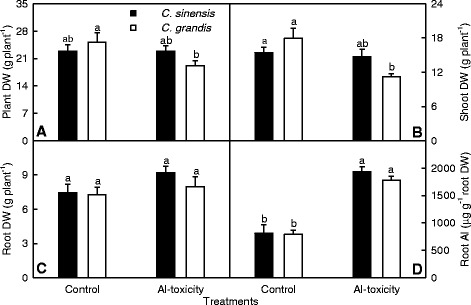
Effects of Al-toxicity on plant growth and root Al concentration in *C. sinensis* and *C. grandis* seedlings. **a-c** Whole plant, shoot and root dry weights (DWs). **d** Root Al concentration. Bar represents the mean ± SE (*n* = 10 for plant DW or 4 for Al concentration). Differences among four treatment combinations were analyzed by 2 (species) × 2 (Al levels) ANOVA. Different letters above the bars indicate a significant difference at *P* < 0.05

As shown in Fig. 1a-c, 1.2 mM Al-treated (+Al) *C. grandis* seedings displayed decreased whole plant and shoot dry weights (DWs) and unchanged root DW, compared with controls, while Al-toxicity did not significantly affect *C. sinensis* whole plant, shoot and root DWs. There was no significant difference in whole plant, shoot and root DWs between the two species at each given Al treatment. Root concentration of Al was higher in + Al seedlings than controls, while did not significantly differ between the two species at each given Al treatment (Fig. 1d).

It is worth mentioning that 1.2 mM Al treatment decreased *C. grandis* root DW, but had no influence on *C. sinensis* root DW and that Al-toxicity-induced decreases in whole plant and shoot DWs were more severe in *C. grandis* than in *C. sinensis* seedlings, when 20 μM H_3_BO_3_ in nutrient solution was replaced by 2.5 μM H_3_BO_3_. In addition, Al concentration was not lower in *C. sinensis* than in *C. grandis* roots at each given Al level (Additional file 1).

### Primary data analysis and protein identification

**Fig. 2:**
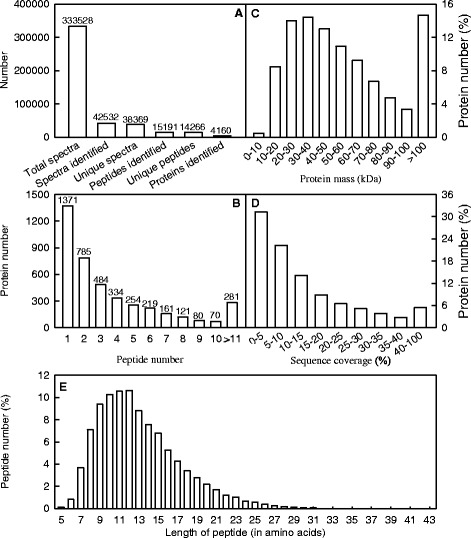
Spectra, peptides and proteins identified from iTRAQ proteomics by searching against *C. sinensis* database (**a**), number of peptides that match to proteins using MASCOT (**b**), protein mass distribution (**c**), distribution of protein sequence coverage (**d**), and distribution of peptide length (**e**)

A total of 333 528 spectra were produced from the iTRAQ experiment using control and Al-toxic *C. sinensis* and *C. grandis* roots as materials. By analyzing these spectra, we identified 42 532 known spectra, 38 369 unique spectra, 15 191 peptides, 14 266 unique peptides and 4160 proteins, respectively (Fig. 2a). The number of proteins in the genome is 46147 (http://phytozome.jgi.doe.gov/pz/portal.html#!info?alias=Org_Csinensis), due to the reasons of samples and technical restrain, 4160 identified proteins in current proteomic research is normal in plants. Protein number decreased with increased number of peptides that matched to proteins, but over 67 % of the proteins (i.e., 2789 proteins) included at least two peptides (Fig. 2b).

Both protein mass distribution and distribution of sequence coverage were summarized in Fig. 2c and d. Proteins with 20–30 kDa and 30–40 kDa were at the most abundant, followed by proteins with 40–50 kDa and 50–60 kDa. Protein number decreased with increased sequence coverage.

Figure 2e shows the distribution of peptide length in *Citrus* roots. Less than 8.7 % of all peptides are ≥ 20 amino acid residues and 80.6 % are between 8 and 17 residues.

### Differentially expressed proteins by iTRAQ

**Fig. 3:**
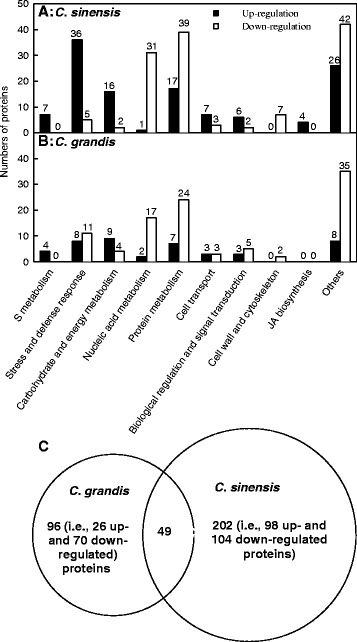
Classification of Al-induced differentially expressed proteins in *C. sinensis* (**a**) and *C. grandis* (**b**) roots and venn diagram analysis of differentially expressed proteins in *Citrus* roots (**c**). Among these overlapping proteins, 45 (4) proteins were regulated in the same (opposite) direction upon Al exposure

**Fig. 4:**
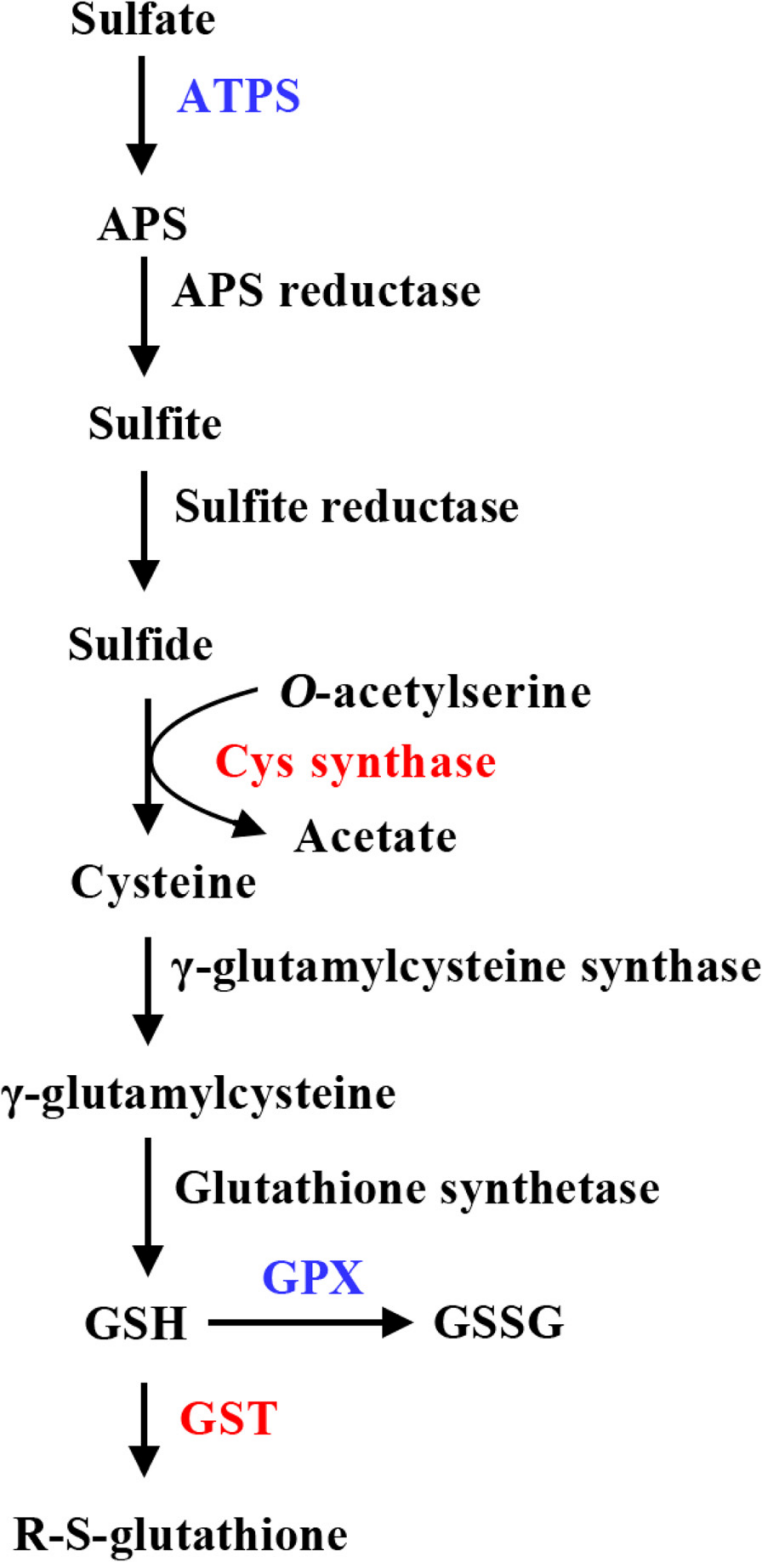
Al-induced changes in S metabolism in *C. sinensis* and *C. grandis* roots. Up-regulated proteins presented in + Al *C. sinensis* and *C. grandis* (only in *C. sinensis*) roots were labeled in red (blue). Abbreviations: APS, adenosine phosphosulphate; GSH, reduced glutathione; GSSG, oxidized glutathione

**Fig. 5:**
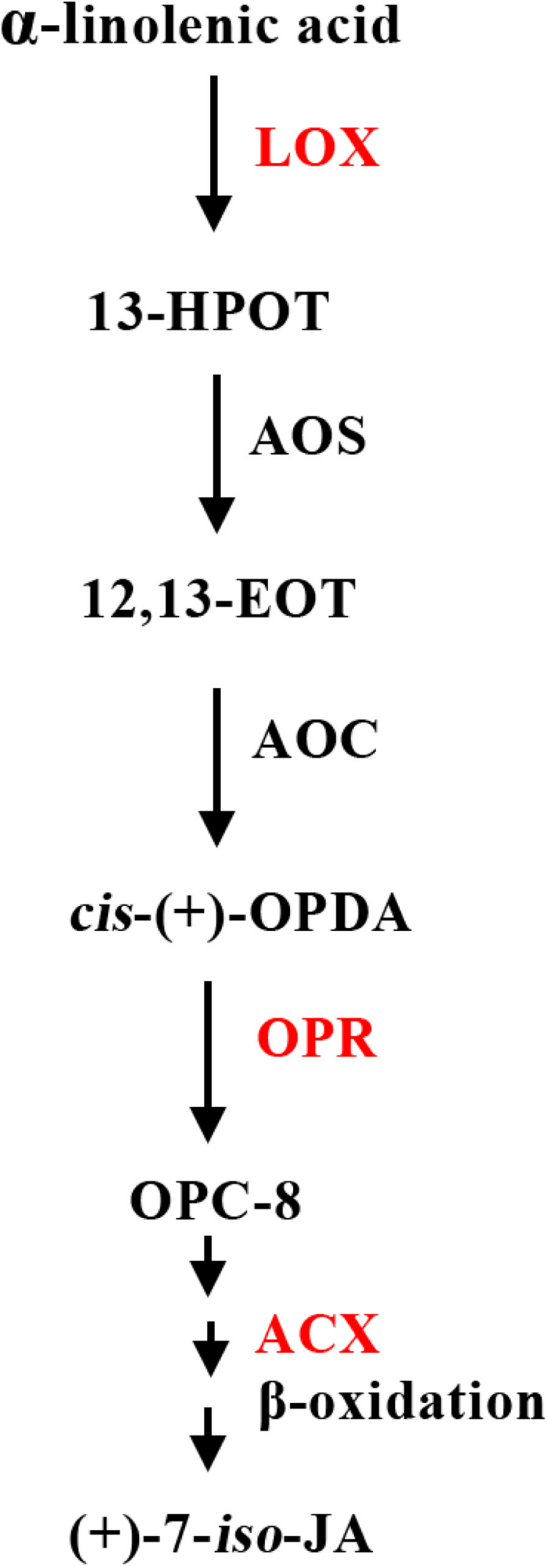
Metabolic scheme of JA biosynthesis in *Citrus sinensis* roots. Up-regulated proteins in + Al roots were labeled in red. Abbreviations: LOX, lipoxygenase; 13-HPOT, (13S)-hydroperoxyoctadecatrienoic acid; AOS, allene oxide synthase; 12,13-EOT, 12,13-epoxyoctadecatrienoic acid; AOC, allene oxide cyclase; *cis*-(+)-OPDA; cis-(+)-12-oxophytodienoic acid; OPR, oxophytodienoic acid reductase; OPC-8, 3-oxo-2-(2′(Z)-pentenyl)-cyclopentane-1-octanoic acid; ACX, acyl-CoA-oxidase

In this study, a protein was considered differentially expressed when the protein had both a log2 fold-change of more than 1.5 and a *P-*value of less than 0.05. Based on the two criteria, 251 differentially expressed proteins were detected in + Al *C. sinensis* roots, 120 (47.8 %) of which displayed increased and 131 (52.2 %) displayed decreased abundance. These proteins were related to sulfur (S) metabolism, stress and defense response, carbohydrate and energy metabolism, nucleic acid metabolism, protein metabolism, cell transport, biological regulation and signal transduction, cell wall and cytoskeleton metabolism, jasmonic acid (JA) biosynthesis and others (Additional file 2 and Fig. 3a). In + Al *C. grandis* roots, we isolated 44 (30.3 %) up- and 101 (69.7 %) down-regulated proteins, which were grouped into following functional categories: S metabolism, stress and defense response, carbohydrate and energy metabolism, nucleic acid metabolism, protein metabolism, cell transport, biological regulation and signal transduction, cell wall and cytoskeleton metabolism and others (Additional file 2 and Fig. 3b). Figures 4 and 5 showed Al-induced alterations of proteins involved in S metabolism in *C. sinensis* and *C. grandis* roots and JA biosynthesis in *C. sinensis* roots, respectively.

As shown in Fig. 3c, a total of 347 differentially expressed proteins were detected from + Al *C. grandis* and *C. sinensis* roots. Among these proteins, 202 (96) proteins only presented in *C. sinensis* (*C. grandis*) and 49 proteins were shared by the two species. Of the 49 overlapping proteins, 45 proteins were regulated in the same direction upon Al exposure in the both species, the remaining four proteins were regulated in the opposite direction.

### qRT-PCR analysis of genes for some differentially expressed proteins

**Fig. 6:**
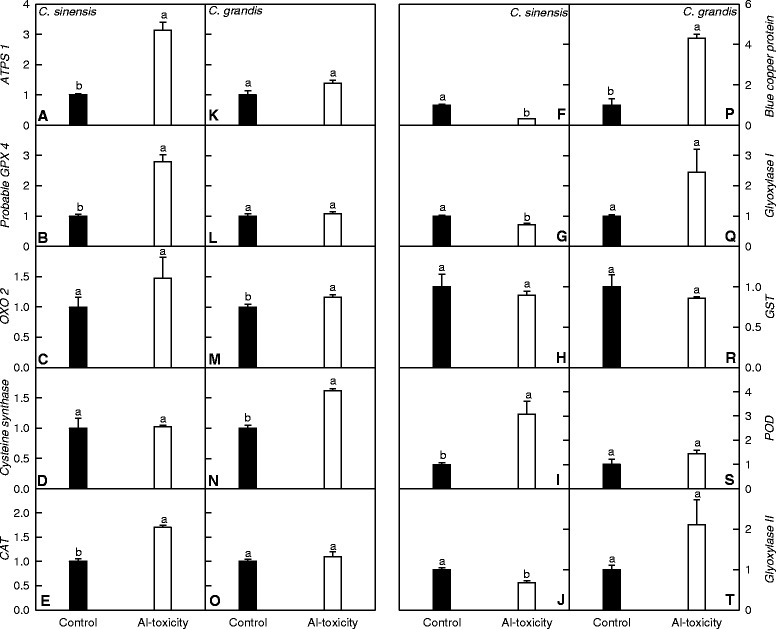
Relative expression levels of genes from *C. sinensis* (**a-j**) and *C. grandis* (**k-t**) roots. Relative abundances of genes encoding ATP sulfurylase 1 (ATPS1, gi|281426908; **a** and **k**), probable glutathione peroxidase 4 (GPX 4, gi|75154467; **b** and **l**), oxalate oxidase 2 (OXO 2, gi|1171937; **c** and **m**), O-acetylserine (thiol)lyase (cysteine synthase), partial (gi|34099833; **d** and **n**), catalase (gi|378724814; CAT, **e** and **o**), blue copper protein (gi|21264375; **f** and **p**), glyoxylase I, partial (gi|301341860; **g** and **q**), glutathione transferase (GST), partial (gi|380863042; **h** and **r**), peroxidase (POD, gi|110007377; **i** and **s**) and hydroxyacylglutathione hydrolase (Glyoxylase II, gi|3913733; **j** and **t**) in Al-toxic and control roots revealed by qRT-PCR. Bar represents the mean ± SE (*n* = 3). Unpaired *t*-test was applied for comparision between means. Different letters above the bars indicate a significant difference at *P* < 0.05. All the values were expressed relative to the control roots

To valuate the correlation between mRNA and protein levels, we assayed the expression levels of genes for 10 differentially expressed proteins (i.e., gi|281426908, gi|75154467, gi|1171937, gi|34099833, gi|378724814, gi|21264375, gi|301341860, gi|380863042, gi|110007377 and gi|3913733) in *C. sinensis* and *C. grandis* roots (Fig. 6). For *C. grandis* roots, the expression levels of nine genes matched well with our iTRAQ data, while only four genes matched well with the iTRAQ data for *C. sinensis* roots (Additional file 2). Six genes [i.e., gi|1171937, gi|34099833, gi|21264375, gi|301341860, gi|380863042 and gi|3913733) were regulated differentially at transcript and protein levels in the both species. Thus, it is reasonable to assume that the discrepancy between the expression levels of genes and the abundances of the corresponding protein might be caused by post-translational modifications (PTMs).

## Discussion

### *C. sinensis* is more tolerant to Al-toxicity than *C. grandis*

Our results showed that Al-toxicity inhibited *C. grandis* shoot growth, but had no significant influence on *C. sinensis* growth, and that root Al concentration did not differ between the two species (Fig. 1). When 20 μM H_3_BO_3_ in nutrient solution was replaced by 2.5 μM H_3_BO_3_, 1.2 mM Al inhibited *C. grandis* root and shoot growth, but only significantly inhibited *C. sinensis* shoot growth even though Al concentration was higher in Al-toxic *C. sinensis* roots than in Al-toxic *C. grandis* ones. Futhermore, Al-toxicity-induced inhibition of shoot growth was less severe in *C. sinensis* seedlings than in *C. grandis* ones (Additional file 1). To conclude, the present work, like that of previous workers [19, 20], demonstrates that *C. sinensis* is more tolerant to Al-toxicity than *C. grandis*.

As shown in Additional file 2, we identified 251 differentially expressed proteins from + Al *C. sinensis* roots, while only 145 differentially expressed proteins were identified from + Al *C. grandis* roots. This demonstrated that the metabolic flexibility was more remarkable in *C. sinensis* roots than in *C. grandis* ones in response to Al, which may contribute to the higher Al-tolerance of *C. sinensis*.

### S metabolism was up-regulated in Al-treated roots

S metabolism is a core pathway for the synthesis of molecules required for some environmental stresses including Al [4, 10]. Transgenic plants over-expressing S metabolism-related genes such as *GST* and *glutathione peroxidase* (*GPX*) displayed enhanced tolerance to Al-toxicity and/or to oxidative stress [21–23]. The up-regulation of ATP sulfurylase 1 (ATPS 1) and probable GPX 4 in *C. sinensis* roots and Cys synthase and GST in *C. sinensis* and *C. grandis* roots in response to Al (Additional file 2 and Fig. 4) indicated that S metabolism was enhanced in Al-treated roots. This agrees with the reports that S metabolism-related enzymes (i.e. GST, GPX, ATPS and Cys synthase) were induced by various stresses including Al [1, 4, 16, 24], and that Al-toxicity increased rice root concentrations of reduced glutathione (GSH) + oxidized glutathione (GSSG) and GSH [4] and *Citrus* leaf concentrations of GSH and GSSG [25]. Thus, S metabolism may play a role in response to Al-toxicity by the synthesis of molecules required for Al detoxification and confer Al-tolerance in both *C. grandis* and *C. sinensis*, particularly in the latter.

### Proteins involved in stress and defense response

Increased reactive oxygen species (ROS) level is commonly presumed to be a major component of Al-toxicity. Here, the abundances of six antioxidant enzymes (i.e., gi|262192812, gi|378724814, gi|255587906, gi|218138216, gi|110007377 and gi|195548074) increased in + Al *C. sinensis* roots (Additional file 2). Al-induced up-regulation of antioxidant enzymes has also been observed in Al-toxic roots of tomato [14] and rice [11]. However, we only identified one up-regulated (i.e., gi|255549391) and two down-regulated (gi|225626263 and gi|89276748) antioxidant enzymes from + Al *C. grandis* ones (Additional file 2). Germin and germin-like proteins (GLPs), which have different enzyme functions such as OXO and SOD, play a role in various abiotic stresses [26, 27]. Study showed that Al-induced OXO expression in wheat roots could be involved in detoxification mechanism [28]. Thus, Al-induced increase in the levels of OXO 2 and seven GLPs in *C. sinensis* roots might play a role in Al-tolerance of *C. sinensis*. However, we only isolated an up-regulated OXO 2 in + Al *C. grandis* roots (Additional file 2). Also, we isolated four up-regulated proteins [i.e., blue copper protein (BCP), CBS domain-containing protein CBSX3, thioredoxin m (mitochondrial)-type, and plastid-lipid associated protein] involved in ROS scavenging from + Al *C. sinensis* roots, while only one up-regulated BCP (gi|21264375) and one down-regulated BCP (gi|2493318) were isolated from + Al *C. grandis* roots (Additional file 2). To sum up, the abundances of proteins involved in ROS detoxification were greatly enhanced in + Al *C. sinensis* roots, but were less affected in + Al *C.grandis* ones.

In addition to ROS formation, Al-toxicity also results in overproduction of aldehydes and methylglyoxal (MG) through the activation of various enzymatic and non-enzymatic reactions. As expected, the abundances of ALDH 7b involved in the detoxification of the aldehydes, alcohol dehydrogenase (ADH)-like 2, which catalyzes the inter-conversions between alcohols and aldehydes, and glyoxalase (Gly) I and Gly II involved in the detoxification of MG were enhanced in Al-toxic *C. sinensis roots*, while the abundance of the probable aldo-keto reductase (AKR) 1 involved in the detoxification of lipid peroxidation- and/or glycolysis-derived reactive carbonyls [i.e., malondialdehyde (MDA), 4-hydoxy-nonenal and MG] was down-regulated in + Al *C. grandis* roots (Additional file 2). Thus, the ability of aldehydes and MG detoxification might be enhanced in + Al *C. sinensis* roots, thus contributing its higher Al-tolerance, but down-regulated in + Al *C. grandis* roots.

The up-regulation of two HSPs (i.e., gi|460411113 and gi|211906498) in + Al *C. sinensis* roots (Additional file 2) agrees with the report that two protein spots of dnaK-type molecular chaperone hsc70.1 (At5g02500) in Al-tolerant *Arabidopsis* ecotype increased in response to Al-toxicity [29], and that the abundance of HSP (LOC_Os06g50300) increased only in Al-tolerant rice roots [11].

Dehydrins, or group 2 LEA (late embryogenesis abundant) proteins, can function as antioxidants, ion sequestrants, or metal ion transporters in plant phloem sap [30]. Al-induced up-regulation of desiccation-related protein PCC13-62 in + Al *C. sinensis* roots (Additional file 2) agrees with the report that root expression level of gene encoding desiccated-related protein under Al-stress was higher in Al-tolerant soybean genotype than in Al-sensitive one [31]. However, Al-toxicity decreased the levels of LEA14-A in *C. sinensis* roots (Additional file 2).

We isolated seven up-regulated (i.e., gi|131026, gi|255587430, gi|21542143, gi|75163188, gi|147721660, gi|7388028 and gi|75115690) and one down-regulated (i.e., gi|190613877) pathogenesis-related (PR) proteins from + Al *C. sinensis* roots, four down-regulated (i.e., gi|190613877, gi|288557882, gi|21542144 and gi|332319679) and one up-regulated (i.e., gi|75115690) PR proteins from + Al *C. grandis* ones. Al-induced increase in S-norcoclaurine synthase (gi|75115690) was higher in *C. sinensis* than in *C. grandis* roots. Generally speaking, Al-toxicity increased the abundances of PR proteins in *C. sinensis* roots and decreased their levels in *C. grandis* roots (Additional file 2). This agrees with the reports that PR-10 protein was highly up-regulated at the transcriptional and translational levels in Al-tolerant soybean cultivar roots [12], that root PR Bet v I family protein (LOC_Os03g18850) were expressed at higher level in the tolerant rice cultivar than in the sensitive one [11], and that the expression levels of several PR genes were higher in Al-tolerant soybean genotype roots than in Al-sensitive ones [31]. Thus, the up-regulation of PR proteins in + Al *C. sinensis* roots might contribute to its Al-tolerance.

Harpin proteins can lead to multiple responses in plants, such as systemic acquired resistance, hypersensitive response, and enhancement of growth and drought tolerance. Over-expression of a harpin-encoding gene in rice increased drought tolerance through ABA signaling [32]. Activated harpin binding protein-1 (HrBP1) can act upstream of the salicylic acid (SA), JA, and ethylene signaling pathways in plant cells [33]. The up-regulation of HrBP1 in + Al *C. sinensis* roots might play a role in Al-tolerance through acting those signaling pathways.

To conclude, the total ability of antioxidation and detoxification was greatly enhanced in + Al *C. sinensis* roots, thus improving the Al-tolerance of *C. sinensis*.

### Proteins involved in carbohydrate and energy metabolism

Rapid turnover rate of fine roots requires systematic rebuilding of tissues, which is associated with a high reduced carbon cost. Building new root biomass not only requires carbon skeletons to produce cellulose, lignin and other structural compounds, it also requires metabolic energy (growth respiration). Under stress conditions, the demand may increase owing to initiation of response mechanisms and secondary metabolism [34]. Thus, carbohydrate and energy metabolism might be enhanced in Al-treated roots to maintain the primary metabolic pathways and the carbohydrate balance. As expected, all the 18 differentially expressed proteins involved in carbohydrate metabolism were up-regulated in + Al *C. sinensis* roots except for two proteins (i.e., gi|75191271 and gi|1351359; Additional file 2). Wang et al. [11] observed that glycolysis/gluconeogenesis was the most significantly up-regulated biochemical process in rice roots in response to Al, suggesting that the enhancement of root glycolytic/gluconeogenetic pathway might promote Al-tolerance by balancing the level of available energy to prevent intracellular shortage. Based on these results, we concluded that the up-regulation of carbohydrate and energy metabolism in + Al *C. sinensis* roots was an adaptive response to meet the increased requirement for carbon skeletons and energy. By contrast, we identified four down- and nine up-regulated proteins involved in carbohydrate and energy metabolism from + Al *C. grandis* roots (Additional file 2). Obviously, Al-induced adaptive responses in carbohydrate and energy metabolism were less pronounced in *C. grandis* than in *C. sinensis* roots.

### Al-induced inhibition of nucleic acid biosynthesis

We found that all these differentially expressed proteins related to nucleic acid metabolism in *C. sinensis* and *C. grandis* roots except for protein RAD-like 3 involved in regulation of transcription in the two species and ribonuclease, which catalyzes the degradation of RNA into smaller components in *C. grandis*, were down-regulated in response to Al-toxicity (Additional file 2), demonstrating that Al-toxicity might impair nucleic acid biosynthesis in *C. sinensis* and *C. grandis* roots and increased RNA degradation in *C. grandis* roots. This agrees with the report that Al-toxicity decreased the concentrations of RNA and DNA, and increased the activities of deoxyribonuclease and ribonuclease in longan (*Dimocarpus longan*) leaves [35].

### Al-induced alteration in protein metabolism

As shown in Additional file 2, all the 39 differentially expressed ribosomal proteins involved in mature ribosome assembly and translation processes were down-regulated in + Al *C. sinensis* (26) and *C. grandis* (13) roots. In addition, the abundances of two proteins (i.e., casein kinase II regulatory subunit and ribosome biogenesis regulatory protein homolog) involved in translation processes, eukaryotic translation initiation factor 5B-like involved in the initiation phase of eukaryotic translation, and peptidyl-prolyl cis-trans isomerase FKBP15-3 involved in protein folding in *C. sinensis* roots, and of two proteins (i.e., probable prefoldin subunit 1 and prefoldin subunit, putative) involved in protein folding decreased in response to Al-stress except for peptidyl-prolyl cis-trans isomerase in *C. sinensis* roots. Therefore, we concluded that Al-toxicity impaired protein biosynthesis in roots. Similar results have been obtained on Al-stressed rice roots [11]. The Al-induced down-regulation of many proteins involved in protein biosynthesis and processing, which are the major consumers of ATP and nutrients, indicated that the translation was reasonably regulated to save energy and nutrients in roots, especially in the Al-tolerant species. Similar results have been obtained on Al-stressed *Arabidopsis* [36] and rice [11] roots. In addition, the widespread down-regulation of root ribosomal proteins, especially in *C. sinensis* roots, might indicate a redeployment of resources to meet the greater demands for amino acids in non-ribosomal peptide synthesis of glutathione and phytochelation for Al complexation. This agrees with the above inference that S metabolism was up-regulated in Al-treated roots, especially in *C. sinensis* roots, and with the reports that Al-toxicity increased rice root concentrations of GSH + GSSG and GSH [4] and *Citrus* leaf concentrations of GSH and GSSG [25].

Protein degradation not only provides molecular substrates for plant respiration but also initiates adaptive responses to (a)biotic stresses by reallocating nutrients from non-essential areas of metabolism to vital cellular activities [37]. As expected, we isolated 13 up-regulated (i.e., two gi|75099392, gi|470138103, gi|257222598, gi|225458529, gi|255538024, gi|332278204, gi|18419649, gi|42563538, gi|416767, gi|33347413, gi|163256765 and gi|353441090) and one down-regulated (i.e., gi|14549156) proteins in proteiolysis from + Al *C. sinensis* roots, five upregulated (i.e., gi|75099392, gi| 75099062, gi|470138103, gi|225458529 and gi|332278204) and two down-regulated (i.e., two gi|14549156 and gi|14549156) proteins in proteolysis from + Al in *C. grandis* roots (Additional file 2). Wang et al. [11] found that the abundance of Cys protease (LOC_Os09g39070) and mitochondrial-processing peptidase subunit (LOC_Os03g11410)] were increased in + Al rice roots, suggesting that Cys protease might play a role in resisting against toxic Al because root Cys protease was expressed at a higher level in the tolerant than in the sensitive cultivar when exposed to Al-toxicity. The more widespread up-regulation of protein in proteolysis in *C. sinensis* than in *C. grandis* roots might contribute to higher Al-tolerance of *C. sinensis*.

Inactive proteins (i.e., incorrect folding) and proteins, which are no longer required for cell, are tagged by ubiquitin for proteolysis. Unlike the above protease, the abundances of all the 11 differentially expressed proteins involved in ubiquitination in *C. sinensis* (6) and *C. grandis* (5) roots decreased in response to Al-toxicity (Additional file 2), meaning that the ubiquitination of some proteins might be impaired in Al-stressed roots.

Six proteins related to amino acid metabolism were altered in + Al *C. sinensis* and *C. grandis* roots (Additional file 2). Al-induced decrease in the abundance of aspartate transaminase in *C. sinensis* roots implied that Al-toxicity might decrease N metabolism flux, because it is the major enzyme controlling aspartate that is used to transport N from sources to sinks [38]. This disagrees with the report that Al-toxicity increased aspartate transaminase level in rice roots, especially in Al-tolerant cultivar [11]. In plants, sarcorsine oxidase metabolizes both sarcosine and pipecolate with preferential utilization of the latter as an endogenous substrate [39]. Here, we first isolated up-regulated sacrosine oxidase in + Al *C. sinensis* roots. Nitrite reductase (NiR) is considered to be a controlling enzyme in plant NO_2_ assimilation. The up-regulation of NiR in + Al *C. sinensis* roots agrees with the report that combined treatment of acidity and Pb^2+^ led to an increase in NiR activity of soybean roots [40]. Phenylalanine ammonia-lyase (PAL) catalyzes the nonoxidative deamination of l-phenylalanine to form trans-cinnamic acid and a free ammonium ion. We observed that Al-toxicity only affected PAL level in Al-intolerant *C. grandis* roots (Additional file 2). However, Wang et al. [11] showed that Al-toxicity decreased PAL abundance in rice roots, especially in Al-tolerant cultivar. Glutamate decarboxylase (GAD) catalyzes the conversion of glutamate to γ-aminobutyrate (GABA). Root-specific GAD (GAD1) is essential for sustaining GABA level in *Arabidopsis* [41]. Therefore, GABA level might be elevated in Al-treated *C. grandis* roots due to enhanced level of putative GAD (Additional file 2). The observed higher level of putative GAD also agrees with the report that rice root *GAD* was expressed primarily under phosphate deprivation [42].

### Proteins involved in cell transport

As shown in Additional file 2, we obtained seven up- and three down-regulated proteins in cell transport from + Al *C. sinensis* roots, and three up- and three down-regulated proteins from + Al *C. grandis* roots. ABC transporter facilitates the movement of S-glutathionylated toxins formed in GST-catalyzed conjugation of GSH to toxins and other substrates across biological membranes [43]. The up-regulation of ABC transporter I family member 17 in + Al *C. sinensis* roots agrees with the increased reguirement for the transport of S-glutathionylated toxins due to increased GST level. Al-induced increase in ABC transporter has been observed in soybean roots [12]. In *Arabidopsis*, a putative ABC transporter-like protein is required for Al-tolerance [44]. The induction of aquaporin in + Al *C. sinensis* roots might be involved in Al-tolerance of *C. sinensis* by regulating the transport of water or micronutrients across cell membranes [45]. However, Wang et al. [11] reported that the abundance of aquaporin PIP2 was down-regulated by ca. 50 % only in Al-tolerant rice cultivar, and that NIP2 in Al-tolerant and -sensitive roots was up-regulated to a similar extent by Al. The up-regulation of porin/voltage-dependent anion-selective channel protein in + Al *C. sinensis* agrees with the report that the abundance of root mitochondrial outer membrane porin 2 (LOC_Os05g45950) increased in Al-tolerant rice cultivar when exposed to Al-toxicity, and decreased in Al-sensitive cultivar [11]. Plasma membrane H^+^-ATPase has an important role in plant response to nutrient and environmental stresses, such as salt stress, Al-stress, P and K deficiencies [46]. Mitochondrial ADP/ATP carrier plays a central role in aerobic cell energetics by providing the ATP generated by oxidative phosphorylation to the cytosol [47]. Sec61, an endoplasmic reticulum (ER) membrane protein translocator, participates in the translocation of newly-synthesized proteins into the lumen of the ER. The hydrolysis of the signal peptide for newly-synthesized proteins is carried out by specific proteases called signal peptidases located on the luminal side of ER. The removal of the signal peptide is crucial for the maintenance of ER homeostasis [48]. The induction of protein transport protein Sec61 subunit β in + Al *C. sinensis* roots might provide an adaptive response by maintaining ER homeostasis. However, its abundance decreased in + Al *C. grandis* roots. By contrast, three proteins (i.e., gi|28380129, gi|289584365 and gi|225463791) in protein transport decreased in + Al *C. sinensis* roots. Besides their role in Fe storage, ferritins also play a role in preventing oxidative damage by storing free Fe in a safe form [49]. Our finding that + Al *C. sinensis* roots had higher abundance of ferritin 1 agrees with the report that there was an increase in the expression of the ferritin Fe storage gene, *AtFER1* in P-deficient *Arabidopsis* leaves [50]. Nucleobase-ascorbate transporters (NATs) have been identified in prokaryotes, fungi, plants and mammals. In plants, maize leaf permease1 (ZmLPE1) has been characterized by functional complementation of a purine transport-deficient *Aspergillus nidulans* strain and is necessary for proper chloroplast development in maize. Interestingly, leaf permease1 protein does not contain a plastidic transit peptide and is expressed only in non-photosynthetic tissues such as roots and etiolated leaves [51]. The up-regulation of putative permease 1 in + Al *C. sinensis* roots might be involved in Al-tolerance throught maintaing chloroplast morphology. Plant lipid transfer proteins (LTPs) are responsible for the shuttling of phospholipids and other fatty acid groups between cell membranes. Some LTPs with broad specificity are termed non-specific LTPs (nsLTPs). We found that the abundances of nsLTP2 and nsLTP-like protein decreased in + Al *C. grandis* roots. This agrees with the reports that nsLTP (E30131) was up-regulated in Al-tolerant cultivar rice roots, and down-regulated in Al-sensitive ones [52], that the expression levels of two genes encoding LTP-like proteins (DT045053 and DT045054) in wheat roots were higher in Al-tolerant than in Al-sensitive near isogenic-line [53], and that mRNA levels of *LTPs* were higher in Al-tolerant than in Al-sensitive soybean genotype [31]. Similarly, putative phosphatidylglycerol/phosphatidylinositol transfer protein DDB_G0282179-like involved in lipid transport was increased in + Al *C. sinensis* roots. However, Wang et al. [11] showed that Al-toxicity decreased the abundance of LTP in Al-tolerance rice roots, but did not signifaicantly affect its level in Al-sensitive ones. Taken all together, the ability of cell transport was enhanced in + Al roots, especially in *C. sinensis*, which might contribute to its higher Al-tolerance.

### Proteins involved in biological regulation and signal transduction

Perception and transmission of stress signals by the cell play crucial roles in plant response to abiotic stresses. Protein kinases and phosphatases are the key players in cell signals. Here, we isolated five up-regulated proteins in phosphorylation (i.e., gi|1174718, gi|388325711, gi|255572716 and gi|255570037) and dephosphorylation (i.e., gi|75248508) in + Al *C. sinensis* roots (Additional file 2), demonstrating that Al-toxicity triggered phosphorylation-dependent signal transduction pathway in *C. sinensis* roots, which might be involved in its Al-tolerance. By contrast, two down-regulated (i.e., gi|6016428 and gi|52077492) and two up-regulated (i.e., gi|33772201 and gi|255558866) proteins in phosphorylation and one up-regulated protein in dephosphorylation (i.e., gi|75248508) were detected in + Al *C. grandis* roots (Additional file 2). This suggested that the balance between phosphorylation and dephosphorylation might be upset and phosphorylation of some proteins might be impaired in + Al *C. grandis* roots. Obviously, *C. sinensis* roots had a higher capacity to keep a better balance between phosphorylation and dephosphorylation than *C. grandis* ones under Al-toxicity, which might contribute to *C. sinensis* Al-tolerance.

Purple acid phosphatases (PAPs) play important roles in P acquisition and recycling in plants [54]. Wang et al. [11] observed that PAP1 expressed at a higher level in Al-tolerant than in Al-sensitive rice root, suggesting that Al-tolerant cultivar might relieve the Al-toxicity through improving the P acquisition. The higher up-regulation of PAP8 in + Al *C. sinensis* than in *C. grandis* roots agrees with our report that the former might more effectively acquire P than the latter with or without Al-stress [19].

Plasma membrane-associated cation-binding protein 1 (PCaP1) is involved in intracellular signals through interaction with phosphatidylinositol phosphates and calmodulin [55]. Our finding that + Al *C. sinensis* roots displayed higher abundance of PCaP1 (Additional file 2) agrees with the report that Cu-stress indced the expression of *PCaP1* in *Arabidopsis* [56].

Our finding that the level of putative G3BP-like protein-like, a protein involved in the Ras signal transduction pathway, was decreased only in + Al *C. grandis* roots (Additional file 2). This agrees with the report that the abundance of G3BP was up-regulated in wild Al-tolerant barley (XZ16) and unchanged in Al-tolerant barley cultivar (Dayton), and down-regulated in Al-sensitive wild barley (XZ61) when exposed to Al-toxicity [15]. These results indicated that the down-regulation of G3BP might be associated with the lower Al-tolerance of *C. grandis*.

### Proteins involved in cell wall and cytoskeleton metabolism

Plant cytoskeleton is highly dynamic networks of proteinaceous components consisting mainly of microtubules and microfilaments. Al-induced growth inhibition and swelling of roots demonstrated that plant cytoskeleton could be a target of Al-toxicity [57]. Studies showed that root microtubules and microfilaments were altered by Al-toxicity [57, 58]. In wheat, Al led to disorganization of actin filaments and formation of actin deposits [59]. As expected, we identified five down-regulated (i.e., gi|14423860, gi|135444, gi|75250086, gi|241865168 and gi|195976596) and two down-regulated (i.e., gi|297600120 and gi|59799374)] proteins related to cytoskeleton metabolism in *C. sinensis* and *C. grandis* roots, respectively. Apart from cytoskeleton proteins, we isolated two down-regulated proteins (i.e., gi|449454512 and gi|224129194) in cell wall metabolism in + Al *C. sinensis* roots (Additional file 2). To conclude, Al-induced alterations in root cell wall and cytoskeleton metabolism differed between the both species.

### JA biosynthesis

Phytohormones play a key role in plant response to (a)biotic stresses. Among these, one of the most important signal molecules is JA [60]. Here, we obtained four up-regulated proteins [i.e., two lipoxygenases (LOXs), long-chain acyl-CoA oxidase (ACX) and 12-oxophytodienoate reductase 2 (OPR2)] involved in JA biosynthesis (Additional file 2; Fig. 5). LOX is one of the key enzymes responsible for the biosynthesis of JA. Al-induced up-regulation of LOX has also been reported in soybean [61] and *Cassia tora* [62, 63] roots. In *Arabidopsis* and rice, OPR3 and OPR 7 are responsible for JA production, respectively [64, 65]. ACX catalyze the first step in the peroxisomal β-oxidation stage of JA biosynthesis. Wound-induced JA accumulation was reduced in a mutant that was defective in ACX1 and was abolished in a mutant that was impaired in both ACX1 and its closely related paralog, ACX5 [66]. Thus, both JA biosynthesis and level might be increased in + Al *C. sinensis* roots, thus enhancing plant Al-tolerance. Because LOX may increase the formation of oxidation products, Al-induced up-regulation of LOX has been suggested to be a response to Al-toxicity [61].

## Conclusions

**Fig. 7:**
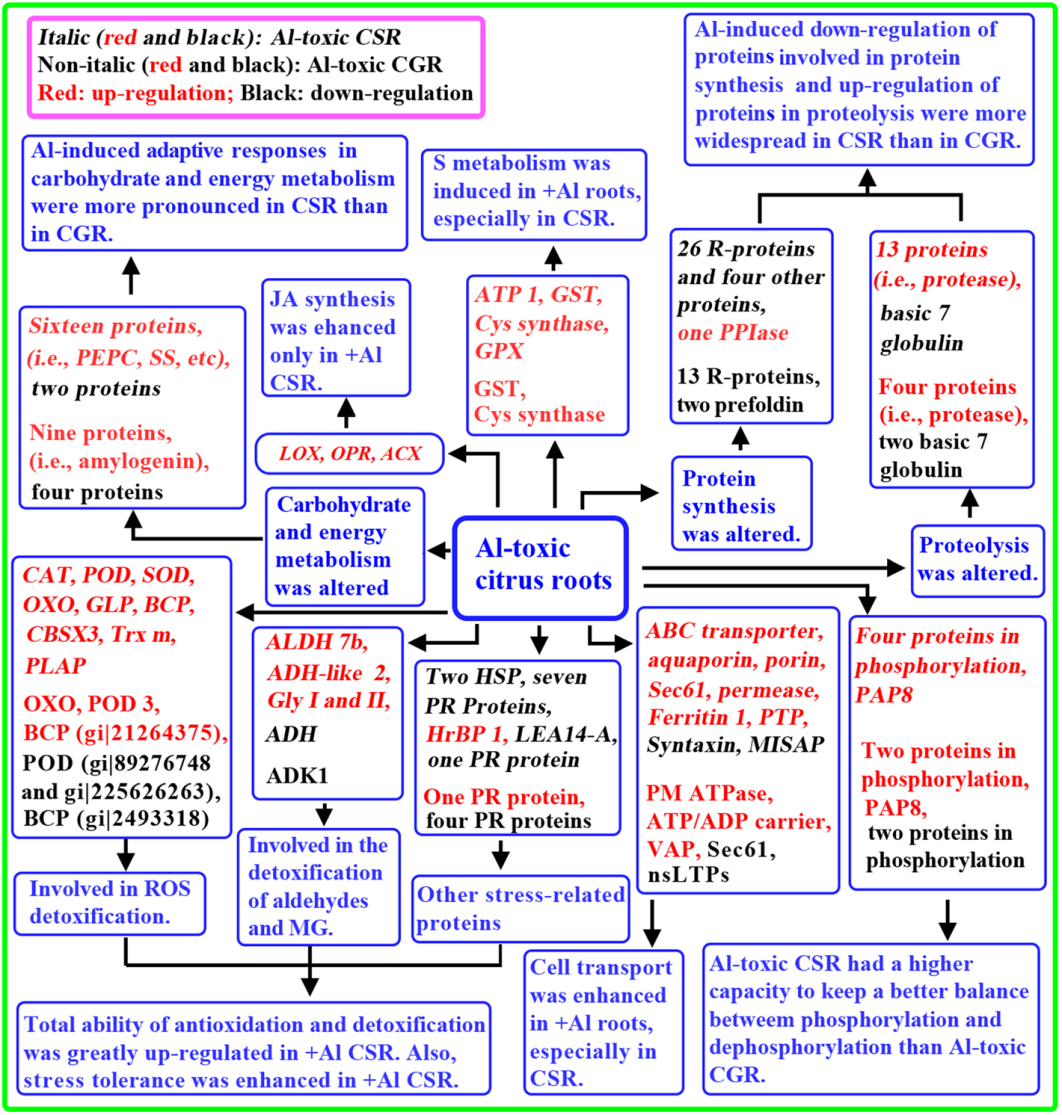
A potential model for the adaptive responses of *C. sinensis* and *C. grandis* roots to Al-toxicity. CGR: *C. grandis* roots; CSR: *C. sinensis* roots; Gly: Glyoxalase; MISAP: Mitochondrial intermembrane space import and assembly protein; PEPC: Phospho*enol*pyruvate carboxylase; PM ATPase: Plasma membrane H^+^-ATPase; PPIase: Peptidyl-prolyl cis-trans isomerase; PLAP: Plastid-lipid-associated protein; R-protein: Ribosomal protein; SS: Sucrose synthase; Trx m: Thioredoxin m; PTP: Phosphatidylglycerol/phosphatidylinositol transfer protein; VAP: Vesicle-associated protein

Our results demonstrate that *C. sinensis* is more tolerant to Al-toxicity than *C. grandis*. Using iTRAQ technique, we isolated 347 differentially expressed proteins from + Al *C. sinensis* and *C. grandis* roots. Among these proteins, 202 proteins only presented in *C. sinensis*, 96 proteins only presented in *C. grandis*, and 49 proteins were shared by the two species. In the 49 overlapping proteins, 45 (4) proteins were regulated in the same (opposite) direction upon Al exposure. This indicated more remarkable metabolic flexibility in *C. sinenis* than in *C. grandis* roots, thus improving the Al-tolerance of *C. sinensis*. As shown in Fig. 7, the higher Al-tolerance of *C. sinensis* might be related to following several factors, including: (*a*) activation of S metabolism; (*b*) greatly improving the total ability of antioxidation and detoxification; (*c*) up-regulation of carbohydrate and energy metabolism; (*d*) enhancing cell transport; (*e*) decreasing (increasing) proteins related to protein synthesis (proteiolysis); (*f*) maintaining a better balance between protein phosphorylation and dephosphorylation; and (*g*) increasing JA biosynthesis. To sum up, we identified more differentially expressed proteins than those of previous studies in other plant roots and provided the most integrated view of the adaptive responses occurring in + Al roots. Therefore, our analysis of root Al-toxicity-responsive proteins will increase our understanding of Al-tolerant mechanisms in higher plants.

## Methods

### Plant culture and Al treatments

This study was conducted from February to December, 2012 at Fujian Agriculture and Forestry University, Fuzhou, China. Both plant culture and Al treatments were performed according to Jiang et al. [67] and Yang et al. [19]. Briefly, five-week-old uniform seedlings of ‘Sour pummelo’ [*Citrus grandis* (L.) Osbeck] and ‘Xuegan’ [*Citrus sinensis* (L.) Osbeck] with a single stem were selected and transplanted to 6 L pots containing fine river sand. Seedlings, two to a pot, were grown in a greenhouse under natural photoperiod. Each pot was supplied with 500 mL of nutrient solution every 2 days. The nutrient solution contained the following macronutrients (in mM): KNO_3_, 1; Ca(NO_3_)_2_,1; KH_2_PO_4_, 0.1; and MgSO_4_, 0.5; and micronutrients (in μM): H_3_BO_3_, 20; MnCl_2_, 2; ZnSO_4_, 2; CuSO_4_, 0.5; (NH_4_)_6_Mo_7_O_24_, 0.065; and Fe-EDTA, 20. Six weeks after transplanting, each pot was supplied daily with a nutrient solution containing 0 (control) or 1.2 mM AlCl_3_ · 6H_2_O (+Al) until the sand was saturated. The pH of the nutrient solutions was adjusted to 4.1 - 4.2 using HCl or NaOH. Eighteen weeks after the beginning of Al treatments, approx. 5-mm-long root apices from new white fibrous roots were excised and immediately frozen in liquid N_2_. Samples were stored at −80 °C until extraction. The remaining seedlings that were not sampled were used to measure whole plant, root and shoot DWs and root Al concentration.

### Plant DWs and Al concentration in roots

At the end of the experiment, 10 plants per treatment from different pots were harvested and divided into shoots (leaves + stems) and roots. Their DWs were measured after being dried at 70 °C for 48 h.

Root Al concentration was determined colorimetrically by the aluminon after being digested in a mixture of HNO_3_ : HClO_4_ [68]. There were six replicates per treatment.

### Protein extraction

Proteins were extracted from frozen roots using a phenol extraction procedure according to Yang et al. [69]. Briefly, equal amounts of frozen root tips from six plants (one plant per pot) were mixed as a biological replicate. There was one biological replicates for each treatment. About 1 g frozen mixed samples were well ground in liquid N_2_ with a mortar and pestle. Four milliliter of ice-cooled buffer containing 100 mM Tris–HCl pH 7.8, 100 mM KCl, 50 mM L-ascorbic acid, 1 % (v/v) Triton X-100, 1 % (v/v) β-mercaptoethanol, and 1 mM phenylmethanesulfonyl fluoride (PMSF) was added to the powder and gently pulverized. The mixture was allowed to thaw slowly on ice. The resulting suspension was transferred to a 10 mL tube, then an equal volume of Tris-phenol (pH 8.0) was added. The mixture was thoroughly vortexed before centrifuging at 13 000 *g* for 15 min at 4 °C. The upper phenolic phase was transferred to a 50 mL tube, and then five volumes of 100 mM ammonium acetate/methanol were added. After being mixed carefully, the mixture was stored at −20 °C overnight. The supernatant was removed carefully after centrifugation at 13 000 g for 15 min at 4 °C, then the protein pellets were suspended in 25 mL of ice-cooled methanol for 2 h at −20 °C. Protein pellets were collected by centrifugation at 13 000 g for 15 min at 4 °C, and then were resuspended in 25 mL of ice-cooled acetone containing 0.1 % β-mercaptoethanol and kept at −20 °C for 2 h. After centrifugation at 13 000 g for 15 min at 4 °C, the pellets were washed twice with 25 mL of ice-cooled acetone, and then dried by lyophilization and finally stored at −80 °C until use. Lyophilized protein samples were grinded to fine powder with pestle and liquid N_2_, then transferred to clean tubes and dissolved in lysis buffer [7 M urea, 2 M thiourea, 4 % 3-((3-cholamidopropyl)dimethylammonio)-1-propanesulfonate (CHAPS), 40 mM Tris–HCl, pH 8.5, 1 mM PMSF and 2 mM EDTA]. After 5 min, 10 mM dithiothreitol (DTT) was added to the samples. The suspension was sonicated at 200 W for 15 min and then centrifuged at 4 °C, 30,000 *g* for 15 min. The supernatant was mixed well with five volumes of chilled acetone containing 10 % (v/v) trichloracetic acid (TCA) and incubated at −20 °C overnight. After centrifugation at 4 °C, 30,000 *g*, the supernatant was discarded. The precipitate was washed with chilled acetone three times. The pellet was air-dried and dissolved in lysis buffer [7 M urea, 2 M thiourea, 4 % Tergitol-type NP40 (NP40), 20 mM Tris–HCl, pH 8.0–8.5]. The suspension was sonicated at 200 W for 15 min and centrifuged at 4 °C, 30,000 *g* for 15 min. The supernatant was transferred to another tube. To reduce disulfide bonds in proteins of the supernatant, 10 mM DTT was added and incubated at 56 °C for 1 h. Subsequently, 55 mM idoacetamide (IAM) was added to block the cysteines, incubated for 1 h in the dark room. The supernatant was mixed well with 55 volumes of chilled acetone for 2 h at −20 °C to precipitate proteins. After centrifugation at 4 °C, 30,000 *g*, the supernatant was discarded, and the pellet was air-dried for 5 min, dissolved in 500 μL 0.5 M tetraethylammonium bromide (TEAB), and sonicated at 200 W for 15 min. Finally, samples were centrifuged at 4 °C, 30,000 *g* for 15 min. The supernatant was transferred to a new tube and quantified using a Bio-Rad Protein Assay kit based on the Bradford method using bovine serum albumin as a standard. The proteins in the supernatant were kept at −80 °C for further analysis.

### iTRAQ analysis and bioinformatic analysis of proteins

iTRAQ analysis was implemented at Beijing Genomics Institute (BGI, Shenzhen, China).

One-hundred μg protein was taken out of Al-toxic and control sample solution and then the protein was digested with Trypsin Gold (Promega, Madison, WI, USA) with the ratio of protein : trypsin = 30: 1 at 37 °C for 16 h. After trypsin digestion, peptides were dried by vacuum centrifugation. Peptides were reconstituted in 0.5 M TEAB and processed according to the manufacture’s protocol for 8-plex iTRAQ reagent (AB Sciex Inc., MA, USA). Briefly, one unit of iTRAQ reagent was thawed and reconstituted in 24 μL isopropanol. Al-toxic and control samples for *C. sinensis* (*C. grandis*) were labeled with 118 and 119 (115 and 117) tags, respectively. The peptides labeled with the isobaric tags were incubated at room temperature for 2 h. The labeled peptide mixtures were then pooled and dried by vacuum centrifugation.

For strong cationic exchange (SCX) chromatography using a Shimadzu LC-20AB HPLC Pump system (Shimadzu Co. Kyoto, Japan), the iTRAQ-labeled peptide mixtures were redissolved in 4 mL of buffer A [25 mM NaH_2_PO_4_ in 25 % acetonitrile (CAN), pH 2.7] and loaded onto a 4.6 × 250 mm Ultremex SCX column. The peptides were eluted at a flow rate of 1 mL min^−1^ with a linear gradient of 5 % buffer B (25 mM NaH_2_PO_4_ and 1 M KCl in 25 % ACN, pH 2.7) for 7 min, 5–60 % buffer B for 20 min and 60–100 % buffer B for 2 min and maintained in 100 % buffer B for 1 min before equilibrating with buffer B for 10 min prior to the next injection. Elution was monitored by measuring the absorbance at 214 nm. The eluted peptides were pooled as 20 fractions, desalted by C-18 coloum and vacuum dried.

Each fraction was resuspended in a certain volume of buffer C (5 % ACN, 0.1 % formic acid) and centrifuged at 20 000 *g* for 10 min. In each fraction, the final concentration of peptide was ca. 0.5 μg μL^−1^. A total of 5 μL of supernatant was loaded onto a Shimadzu LC-20 AD nanoHPLC (Shimadzu Co. Kyoto, Japan) by the autosampler onto a 2 cm C18 trap column (inner diameter, 200 μm), and the peptides were eluted onto a resolving 10 cm analytical C18 column (inner diameter, 75 μm). The samples were loaded at 8 μL min^−1^ for 4 min; then eluted at 300 nL min^−1^ with a linear grandent of 5 % buffer D (95 % ACN, 0.1 % formic acid) for 5 min, 3–35 % buffer D for 35 min, 35–60 % buffer D for 5 min, 60–80 % buffer D for 2 min and maintained in 80 % buffer D for 2 min, finally returned to 5 % buffer D within 1 min and maintained in 5 % buffer D for 10 min.

For the TripleTOF analysis, a TripleTOF 5600 system 5600 (AB SCIEX, Concord, ON, Canada) fitted with a Nanospray III source and a pulled quartz tip as the emitter was used. Data were acquired using an ion spray voltage of 2.5 kV, N gas of 30 psi, nebulizer gas of 15 psi, and an interface heater temperature of 150 °C. The MS was operated with an RP of ≥30,000 FWHM for TOF-MS scans. For information dependent acquisition (IDA), survey scans were acquired in 250 ms and as many as 30 product ion scans were collected if they exceeded a threshold of 120 cps with a 2^+^ to 5^+^ charge-state. The total cycle time was fixed to 3.3 s and the Q2 transmission window was 100 Da for 100 %. Four time bins were summed for each scan at a pulser frequency value of 11 kHz through monitoring of the 40 GHz multichannel TDC detector with four-anode/channel detection. A sweeping collision energy setting of 35 ± 5 eV coupled with iTRAQ adjust rolling collision energy was applied to all precursor ions for collision-induced dissociation. Dynamic exclusion was set for 1/2 of peak width (15 s), and then the precursor was refreshed off the exclusion list.

Raw data files acquired from the TripleTof were converted into MGF files using Proteome Discoverer 1.2 and the MGF files were searched. Proteins identification was performed by using Mascot search engine (Version 2.3.02; Matrix Science, London, UK) against *C. sinensis* database (http://www.phytozome.net/cgi-bin/gbrowse/citrus/). For protein identification, a mass tolerance of ±0.05 Da was permitted for intact peptide masses and ±0.1 Da for fragmented ions, with allowance for one missed cleavages in the trypsin digests. Pyrophosphorylation of glutamine and variable oxidation of methionine and iTRAQ labeling of tyrosine were set as variable modification; carbamidomethylation of Cys and iTRAQ labeling of lysine the N-terminal amino group of peptides were set as fixed modification. The peptide charge was set as *Mr*, and monoisotopic mass was chosen. To decrease the probability of false peptide identification, only peptides with significance scores (≥20) at the 99 % confidence interval by a Mascot probability analysis greater than “identity” were counted as identified. Each confident protein identification included at least one unique peptide. An automatic decoy database search strategy was used to estimate the false discovery rate (FDR), which was calculated as the false positive matches divided by the total matches. In the final search results, the FDR was less than 1.5 %. The iTRAQ 8-plex was chosen for quantification during the search.

The search results were filtered before data exportation. The filters were used for protein identification with these options: significance threshold *P* < 0.05 (with 95 % confidence) and ion score or expected cut-off less than 0.05 (with 95 % confidence). For protein quantitation, the filters were set as follows: (*a*) “median” was chosen for the protein ratio type; (*b*) the minimum precursor charge was set to 2 and minimum peptides were set to 2, and only unique peptides were used for quantitation; and (*c*) normalization by median intensities, and outliers were removed automatically. The peptide threshold was set as above for identity. A 1.5 log2-fold change was set to identify significant differentially expressed proteins in addition with a *P-*value of less than 0.05. Distribution fitting of protein ratios data was performed using SPSS software. The two protein datasets were fitted into Normal distribution models and the threshold for significance of +/− 1.5 was sufficient to separate differentially expressed proteins.

Bioinformatic analysis of proteins was performed according to Yang et al. [69].

### Quantitative RT-PCR (qRT-PCR) analysis of gene expression

Root tips of six plants from different pots were mixed as a biological replicate. Equal amounts of root tips were collected from each plant. There were three biological replicates for each treatment. Total RNAs were independently extracted three times from the frozen roots of Al-toxic and control plants using Recalcirtant Plant Total RNA Extraction Kit (Centrifugal column type, Bioteke Corporation, China) according to manufacturer’s instructions. Gene-specific primers were designed using Primer Software Version 5.0 (PREMIER Biosoft International, CA, USA) according to the corresponding sequences of selected proteins in *Citrus* genome (http://www.phytozome.net/cgi-bin/gbrowse/citrus/). The sequences of the F and R primers used are given in Additional file 3. qRT-PCR analysis was performed according to Zhou et al. [70]. For the normalization of gene expression, β-tubulin (JN580571) gene was used as an internal standard and the roots from control plants were used as reference sample, which was set to 1.

### Experimental design and statistical analysis

There were 40 seedlings (20 pots) in a completely randomized design. Experiments were performed with 3–10 replicates except for iTRAQ analysis (i.e., one biological replicate). The replicates represented material from individual plant except for iTRAQ and qRT-PCR analysis [i.e., each biological replicate was created by pooling equal roots from six different plants (one plant per pot)]. Differences among four treatment combinations were analyzed by 2 (species) × 2 (Al levels) ANOVA. The unpaired *t*-test was applied for comparison between two means (i.e., qRT-PCR data). Power analysis was performed by using pwr.t.test functions of pwr.package in R environment (Version 3.2.0). Under empirical μ and σ, the minimum sample size of 8 and 3 could generate a power value of more than 0.8 in plant DW measurement and the other experiments, respectively.

### Availability of supporting data

The mass spectrometry proteomics data have been deposited to the ProteomeXchange Consortium via the PRIDE partner repository with the dataset identifier PXD002301.

## Additional files

Additional file 1:
**Effects of Al-toxicity on plant growth and root Al concentration in** ***Citrus sinensis*** **and** ***C. grandis*** **seedlings.** (DOC 184 kb) Additional file 2:
**List of differentially expressed proteins in Al-toxicity** ***Citrus sinensis*** **(CS) and** ***C. grandis*** **(CG) roots.** (DOC 418 kb) Additional file 3:
**Specific primer pairs used for qRT-PCR expression analysis.** (DOC 40 kb)

## Abbreviations

12,13-EOT: 12,13-epoxyoctadecatrienoic acid
13-HPOT: (13S)-hydroperoxyoctadecatrienoic acid
2-DE: Two-dimensional gel electrophoresis
ABC: ATP Binding Cassette
ACC: 1-aminocydopropane-1-carboxylate
ACX: Aacyl-CoA-oxidase
ADH: Alcohol dehydrogenase
AKR: Aldo-keto reductase
Al: Aluminum
ALDH: Aldehyde dehydrogenase
AOC: Allene oxide cyclase
AOS: Allene oxide synthase
APX: Ascorbate peroxidase
ATPS: ATP sulfurylase
BCP: Blue copper protein
CAT: Catalase
cis-(+)-OPDA: cis-(+)-12-oxophytodienoic acid
Cu/Zn SOD: Copper/zinc superoxide dismutase
DHAR: dehydroascorbate reductase
DW: Dry weight
GAD: Glutamate decarboxylase
GLP: Germin and germin-like protein
Gly: Glyoxalase
GPX: Glutathione peroxidase
GR: Glutathione reductase
GSH: Reduced glutathione
GSSG: Oxidized glutathione
GST: Glutathione S-transferase
HSP: Heat shock protein
iTRAQ: Isobaric tags for relative and absolute quantification
JA: Jasmonic acid
LEA: Late embryogenesis abundant protein
LOX: Lipoxygenase
LTP: Lipid transfer protein
MDA: Malondialdehyde
MDH: Malate dehydrogenase
MG: Methylglyoxal
MLP: Major latex protein
NAT: Nucleobase-ascorbate transporter
NiR: Nitrite reductase
nsLTP: Non-specific LTP
OPC-8: 3-oxo-2-(2'(Z)-pentenyl)-cyclopentane-1-octanoic acid
OPR: Oxophytodienoic acid reductase
OXO: Oxalate oxidase
PAL: Phenylalanine ammonia-lyase
PAP: Purple acid phosphatase
POD: Peroxidase
PTM: Post-translational modification
ROS: reactive oxygen species
S: sulfur
SA: Salicylic acid
