# Supplementary material for: Root iTRAQ protein profile analysis of two Citrus species differing in aluminum-tolerance in response to long-term aluminum-toxicity
Source: BMC Genomics. 2015 Nov 16;16:949. doi: 10.1186/s12864-015-2133-9 (PMC4647617; doi:10.1186/s12864-015-2133-9)
Supplement: Additional file 1: — Effects of Al-toxicity on plant growth and root Al concentration in Citrus sinensis and C. grandis seedlings. (DOC 184 kb) [file 12864_2015_2133_MOESM1_ESM.doc]

**Additional file 1: Figure S1. Effects of Al-toxicity on plant growth and root Al concentration in** ***Citrus sinensis* and** ***C. grandis* seedlings.** **(A-C)** Whole plant, shoot and root dry weights (DWs). **(D)** Root Al concentration. Seedlings were treated with nutrient solution containing 1 mM KNO_3_, 1 mM Ca(NO_3_)_2_, 0.1 mM KH_2_PO_4_, 0.5 mM MgSO_4_, 20 μM H_3_BO_3_, 2 μM MnCl_2_, 2 μM ZnSO_4_, 0.5 μM CuSO_4_, 0.065 μM (NH_4_)_6_Mo_7_O_24_, 20 μM Fe-EDTA, with or without 1.2 mM AlCl_3_·6H_2_O for 18 weeks. Bar represents the mean ± SE (n = 10 for plant DW or 4 for Al concentration). Differences among four treatments were analyzed by 2 (species) × 2 (Al levels) ANOVA. Different letters above the bars indicate a significant difference at *P* < 0.05.
