# Supplementary material for: Root iTRAQ protein profile analysis of two Citrus species differing in aluminum-tolerance in response to long-term aluminum-toxicity
Source: BMC Genomics. 2015 Nov 16;16:949. doi: 10.1186/s12864-015-2133-9 (PMC4647617; doi:10.1186/s12864-015-2133-9)
Supplement: Additional file 2: — List of differentially expressed proteins in Al-toxicity Citrus sinensis (CS) and C. grandis (CG) roots. (DOC 418 kb) [file 12864_2015_2133_MOESM2_ESM.doc]

**Additional file 2** List of differentially expressed proteins in Al-toxicity *Citrus sinensis* (CS) and *C. grandis* (CG) roots.

| **Accession** | **Homology** | **Protein** | **Fold change** | |
| --- | --- | --- | --- | --- |
|  |  |  | **CS**  **(118/119)** | **CG**  **(115/117)** |
| ***S metabolism*** |  |  |  |  |
| orange1.1g012433m|PACid:18106261 | gi|281426908 | ATP sulfurylase 1 | 1.715 |  |
| **orange1.1g020528m|PACid:18133307** | **gi|34099833** | **O-acetylserine (thiol)lyase, partial (Cysteine synthase)** | **1.618** | **1.518** |
| orange1.1g036444m|PACid:18116690 | gi|255568460 | Glutathione-s-transferase omega, putative | 2.256 |  |
| **orange1.1g027333m|PACid:18135571** | **gi|380863046** | **Glutathione transferase, partial** | **2.308** | **2.101** |
| orange1.1g045074m|PACid:18116693 | gi|109630981 | Glutathione-S-transferase | 3.684 |  |
| **orange1.1g042301m|PACid:18120525** | **gi|380863042** | **Glutathione transferase, partial** | **1.777** | **1.632** |
| orange1.1g030845m|PACid:18093214 | gi|75154467 | Probable glutathione peroxidase 4 | 1.507 |  |
| orange1.1g041305m|PACid:18101597 | gi|75263009 | Glutathione S-transferase U17 |  | 1.537 |
|  |  |  |  |  |
| ***Stress and defense response*** |  |  |  |  |
| orange1.1g044386m|PACid:18092254 | gi|262192812 | Catalase | 1.573 |  |
| orange1.1g042356m|PACid:18092056 | gi|378724814 | Catalase | 4.546 |  |
| orange1.1g018873m|PACid:18128426 | gi|255587906 | Peroxidase C3 precursor, putative | 1.975 |  |
| orange1.1g026199m|PACid:18119146 | gi|195548074 | Iron superoxide dismutase | 1.658 |  |
| orange1.1g019278m|PACid:18091383 | gi|218138216 | Peroxidase 4 | 1.670 |  |
| orange1.1g018811m|PACid:18128473 | gi|110007377 | Peroxidase | 1.852 |  |
| **orange1.1g040384m|PACid:18119464** | **gi|1171937** | **Oxalate oxidase 2** | **1.931** | **1.804** |
| orange1.1g027375m|PACid:18132452 | gi|75228449 | Germin-like protein 1-1 | 2.344 |  |
| orange1.1g027919m|PACid:18129993 | gi|351723525 | Germin-like protein 21 | 1.637 |  |
| orange1.1g027622m|PACid:18119236 | gi|15241653 | Germin-like protein subfamily 1 member 17 | 2.128 |  |
| orange1.1g027345m|PACid:18119233 | gi|393008685 | Germin-like protein | 2.460 |  |
| orange1.1g045405m|PACid:18132485 | gi|75228449 | Germin-like protein 1-1 | 2.172 |  |
| orange1.1g037035m|PACid:18132232 | gi|18203242 | Germin-like protein subfamily T member 2 | 2.851 |  |
| orange1.1g027369m|PACid:18119229 | gi|15241653 | Germin-like protein subfamily 1 member 17 | 1.736 |  |
| orange1.1g042226m|PACid:18114381 | gi|225447031 | PREDICTED: putative germin-like protein 2-1 | 1.949 |  |
| **orange1.1g042021m|PACid:18116199** | **gi|21264375** | **Blue copper protein** | **3.199** | **2.808** |
| orange1.1g028667m|PACid:18119362 | gi|20455364 | CBS domain-containing protein CBSX3, mitochondrial | 1.677 |  |
| orange1.1g030784m|PACid:18100172 | gi|255555435 | Thioredoxin m (mitochondrial)-type, putative | 1.653 |  |
| orange1.1g020639m|PACid:18112288 | gi|3928760 | Plastid-lipid-associated protein, chloroplastic | 1.668 |  |
| orange1.1g010520m|PACid:18132980 | gi|375152084 | Aldehyde dehydrogenase 7b, partial | 1.563 |  |
| orange1.1g017201m|PACid:18139634 | gi|75337342 | Alcohol dehydrogenase-like 2 | 1.590 |  |
| orange1.1g016933m|PACid:18131872 | gi|1351887 | Alcohol dehydrogenase | 0.527 |  |
| orange1.1g034154m|PACid:18129485 | gi|108936030 | Putative allyl alcohol dehydrogenase-like protein | 0.599 |  |
| orange1.1g026030m|PACid:18104367 | gi|301341860 | Glyoxylase I, partial | 1.843 |  |
| orange1.1g027699m|PACid:18095456 | gi|3913733 | Hydroxyacylglutathione hydrolase cytoplasmic (Glyoxylase II) | 1.566 |  |
| orange1.1g036208m|PACid:18097289 | gi|460411113 | Heat shock cognate 70 kDa protein-like | 2.003 |  |
| orange1.1g020168m|PACid:18110977 | gi|211906498 | Heat shock protein 70 | 1.655 |  |
| orange1.1g021580m|PACid:18136940 | gi|118926 | Desiccation-related protein PCC13-62 | 1.635 |  |
| orange1.1g031863m|PACid:18108589 | gi|1170745 | Late embryogenesis abundant protein Lea14-A | 0.488 |  |
| orange1.1g031343m|PACid:18109345 | gi|131026 | Pathogenesis-related protein STH-2 | 1.605 |  |
| orange1.1g041437m|PACid:18129081 | gi|255587430 | Major latex protein, putative | 2.667 |  |
| orange1.1g039477m|PACid:18095861 | gi|21542143 | MLP-like protein 34 | 2.529 |  |
| orange1.1g031888m|PACid:18094550 | gi|75163188 | MLP-like protein 423 | 1.534 |  |
| orange1.1g044703m|PACid:18103025 | gi|147721660 | Major pollen allergen Que a 1 (Fragment) | 2.727 |  |
| orange1.1g036858m|PACid:18109651 | gi|7388028 | Major allergen Pru av 1 | 1.524 |  |
| **orange1.1g031668m|PACid:18093713** | **gi|75115690** | **S-norcoclaurine synthase** | **3.008** | **2.396** |
| **orange1.1g031459m|PACid:18109810** | **gi|190613877** | **Putative allergen Pru p 1.02** | **0.256** | **0.500** |
| **orange1.1g020203m|PACid:18128349** | **gi|3337091** | **Polygalacturonase inhibitor (PGIP)** | **1.740** | **1.605** |
| orange1.1g041139m|PACid:18106708 | gi|38679311 | Harpin binding protein 1 | 1.968 |  |
| orange1.1g048139m|PACid:18092663 | gi|122196004 | Elicitor-responsive protein 3 (16 kDa phloem protein) | 0.629 |  |
| **orange1.1g022993m|PACid:18102444** | **gi|75271990** | **Hypersensitive-induced response protein 2** | **1.816** | **1.515** |
| orange1.1g020084m|PACid:18104526 | gi|225626263 | Peroxidase |  | 0.654 |
| orange1.1g020951m|PACid:18139265 | gi|89276748 | Peroxidase |  | 0.635 |
| orange1.1g048664m|PACid:18094570 | gi|255549391 | Peroxidase 3 precursor, putative |  | 1.569 |
| orange1.1g030729m|PACid:18139547 | gi|2493318 | Blue copper protein |  | 0.649 |
| orange1.1g020083m|PACid:18119714 | gi|378548275 | Probable aldo-keto reductase 1 |  | 0.400 |
| orange1.1g031431m|PACid:18109390 | gi|288557882 | Pathogenesis-related protein 10.5 |  | 0.537 |
| orange1.1g031775m|PACid:18095795 | gi|21542144 | MLP-like protein 28 |  | 0.413 |
| orange1.1g034001m|PACid:18095657 | gi|332319679 | Kirola |  | 0.619 |
| orange1.1g033309m|PACid:18112501 | gi|119367470 | Putative auxin-repressed/dormancy-associated protein |  | 0.625 |
| orange1.1g033104m|PACid:18134808 | gi|195640348 | Auxin-repressed protein |  | 0.644 |
| orange1.1g027801m|PACid:18128637 | gi|296084377 | 6,7-dimethyl-8-ribityllumazine synthase (lumazine synthase) |  | 2.116 |
| orange1.1g031202m|PACid:18114994 | gi|224077455 | Predicted protein |  | 0.547 |
| orange1.1g046228m|PACid:18134165 | gi|225427027 | 14 kDa proline-rich protein DC2.15 isoform 1 |  | 1.769 |
|  |  |  |  |  |
| ***Carbohydrate and energy metabolism*** |  |  |  |  |
| orange1.1g017992m|PACid:18135302 | gi|462412655 | Aldose 1-epimerase | 1.553 |  |
| orange1.1g047621m|PACid:18094947 | gi|62321271 | Beta-xylosidase | 1.592 |  |
| orange1.1g001537m|PACid:18121670 | gi|73917652 | Phospho*enol*pyruvate carboxylase 4 | 1.556 |  |
| orange1.1g005865m|PACid:18107705 | gi|371940831 | Phospho*enol*pyruvate carboxykinase, partial | 1.749 |  |
| orange1.1g006486m|PACid:18124585 | gi|371939985 | Phospho*enol*pyruvate carboxykinase, partial | 2.260 |  |
| orange1.1g012041m|PACid:18133646 | gi|255541894 | Enolase, putative | 1.515 |  |
| orange1.1g009516m|PACid:18133441 | gi|255577875 | Succinate semialdehyde dehydrogenase, putative | 1.783 |  |
| orange1.1g035501m|PACid:18092452 | gi|313295 | Branching enzyme, partial | 2.173 |  |
| orange1.1g003492m|PACid:18136627 | gi|6682841 | Sucrose synthase | 1.942 |  |
| **orange1.1g010588m|PACid:18102692** | **gi|357475019** | **Beta-glucosidase** | **2.615** | **1.717** |
| orange1.1g011765m|PACid:18139297 | gi|75249511 | UDP-glycosyltransferase 91A1 | 1.603 |  |
| orange1.1g040435m|PACid:18099309 | gi|357466355 | Alpha-1,4-galacturonosyltransferase | 1.592 |  |
| orange1.1g026510m|PACid:18105917 | gi|8928122 | Endo-1,3;1,4-beta-D-glucanase | 1.652 |  |
| orange1.1g002036m|PACid:18103793 | gi|357456317 | Lysosomal alpha-mannosidase | 2.060 |  |
| orange1.1g011636m|PACid:18126076 | gi|38257732 | Glucan endo-1,3-beta-glucosidase (beta-1,3-glucanase) 6 | 1.632 |  |
| **orange1.1g042606m|PACid:18124258** | **gi|75165175** | **NADH dehydrogenase [ubiquinone] 1 beta subcomplex subunit 10-B** | **1.537** | **0.614** |
| orange1.1g034182m|PACid:18116464 | gi|75191271 | NADH dehydrogenase [ubiquinone] iron-sulfur protein 6, mitochondrial | 0.473 |  |
| orange1.1g034047m|PACid:18092126 | gi|1351359 | Cytochrome b-c1 complex subunit 6 | 0.603 |  |
| orange1.1g009075m|PACid:18136893 | gi|223635599 | Probable polygalacturonase |  | 0.621 |
| orange1.1g018955m|PACid:18093650 | gi|2274915 | Beta-1,3-glucanase |  | 0.594 |
| orange1.1g034138m|PACid:18130606 | gi|75206429 | NADH dehydrogenase [ubiquinone] 1 beta subcomplex subunit 7 |  | 0.608 |
| orange1.1g042753m|PACid:18133610 | gi|255547071 | UDP-glucosyltransferase, putative |  | 1.760 |
| orange1.1g008722m|PACid:18114375 | gi|20455195 | Pectinesterase |  | 1.718 |
| orange1.1g008242m|PACid:18132945 | gi|75102977 | Beta-fructofuranosidase, insoluble isoenzyme |  | 1.550 |
| orange1.1g024846m|PACid:18122316 | gi|255576607 | (S)-2-hydroxy-acid oxidase, putative |  | 1.537 |
| orange1.1g039466m|PACid:18095998 | gi|255576603 | (S)-2-hydroxy-acid oxidase, putative |  | 1.519 |
| orange1.1g021904m|PACid:18112922 | gi|319739583 | Putative L-galactose-1-phosphate phosphatase |  | 1.556 |
| orange1.1g039651m|PACid:18122527 | gi|2130118 | Amylogenin - maize (fragments) |  | 1.757 |
| orange1.1g034238m|PACid:18124636 | gi|75213718 | Cytochrome c oxidase subunit 6a, mitochondrial |  | 1.569 |
|  |  |  |  |  |
| ***Nucleic acid metabolism*** |  |  |  |  |
| orange1.1g006380m|PACid:18096179 | gi|355491566 | Splicing factor-like protein | 0.621 |  |
| orange1.1g009052m|PACid:18124713 | gi|75338884 | Splicing factor U2af large subunit B | 0.643 |  |
| **orange1.1g022930m|PACid:18107318** | **gi|195612902** | **Splicing factor, arginine/serine-rich 2** | **0.220** | **0.515** |
| **orange1.1g025401m|PACid:18128541** | **gi|255582554** | **Serine/arginine rich splicing factor, putative** | **0.361** | **0.522** |
| **orange1.1g021599m|PACid:18095318** | **gi|470143569** | **Serine/arginine-rich splicing factor RS2Z32-like isoform 2** | **0.529** | **0.535** |
| **orange1.1g041602m|PACid:18102432** | **gi|449434905** | **LOW QUALITY PROTEIN: serine/arginine-rich splicing factor RSZ21-like** | **0.571** | **0.582** |
| orange1.1g022301m|PACid:18137163 | gi|255564164 | Arginine/serine-rich splicing factor, putative | 0.661 |  |
| orange1.1g046599m|PACid:18109183 | gi|357018296 | SR-protein | 0.597 |  |
| orange1.1g035838m|PACid:18109185 | gi|6226648 | DNA mismatch repair protein MSH6 | 0.514 |  |
| orange1.1g014614m|PACid:18128622 | gi|356531876 | Putative RNA-binding protein Luc7-like 2-like | 0.580 |  |
| **orange1.1g026421m|PACid:18098867** | **gi|355523249** | **ELAV-like protein** | **0.470** | **0.611** |
| orange1.1g045516m|PACid:18129341 | gi|75325377 | Nucleolin 1 | 0.536 |  |
| orange1.1g039373m|PACid:18127859 | gi|21362538 | Probable rRNA-processing protein EBP2 homolog | 0.483 |  |
| **orange1.1g038197m|PACid:18101083** | **gi|20532086** | **Putative DNA-binding protein ESCAROLA** | **0.463** | **0.660** |
| orange1.1g031973m|PACid:18098560 | gi|123378 | DNA-binding protein MNB1B | 0.646 |  |
| **orange1.1g022395m|PACid:18099851** | **gi|73919939** | **Histone deacetylase HDT1** | **0.493** | **0.497** |
| orange1.1g031615m|PACid:18137023 | gi|225453837 | Histone deacetylase complex subunit SAP18 | 0.646 |  |
| orange1.1g022827m|PACid:18115204 | gi|356569696 | THO complex subunit 4-like | 0.641 |  |
| orange1.1g015459m|PACid:18115448 | gi|75283672 | Transcription factor bHLH155 | 0.595 |  |
| **orange1.1g000847m|PACid:18121691** | **gi|26397174** | **Putative nuclear matrix constituent protein 1-like protein** | **0.589** | **0.520** |
| orange1.1g022880m|PACid:18117949 | gi|75228574 | Zinc finger CCCH domain-containing protein 14 | 0.653 |  |
| orange1.1g005082m|PACid:18101721 | gi|122166929 | Zinc finger CCCH domain-containing protein 53 | 0.605 |  |
| orange1.1g005338m|PACid:18114251 | gi|190899694 | C-x8-C-x5-C-x3-H type Zn-finger | 0.516 |  |
| orange1.1g028027m|PACid:18113168 | gi|359487818 | MKI67 FHA domain-interacting nucleolar phosphoprotein-like | 0.642 |  |
| orange1.1g008794m|PACid:18123570 | gi|224121958 | Nucleolar family protein | 0.554 |  |
| orange1.1g026902m|PACid:18104675 | gi|357461607 | U1 small nuclear ribonucleoprotein A | 0.636 |  |
| orange1.1g008852m|PACid:18120230 | gi|147744575 | Probable nucleolar protein 5-1 | 0.424 |  |
| orange1.1g003362m|PACid:18095419 | gi|449451683 | U4/U6 small nuclear ribonucleoprotein Prp3-like | 0.634 |  |
| orange1.1g007467m|PACid:18103992 | gi|255619323 | Centromere/microtubule binding protein cbf5, putative | 0.422 |  |
| **orange1.1g005235m|PACid:18094325** | **gi|354551239** | **WD40 repeat protein** | **0.574** | **0.639** |
| orange1.1g012070m|PACid:18123410 | gi|255549595 | Bystin, putative | 0.639 |  |
| **orange1.1g041475m|PACid:18092359** | **gi|240256186** | **Protein RAD-like 3** | **2.469** | **3.201** |
| orange1.1g043937m|PACid:18133757 | gi|544421 | Glycine-rich RNA-binding protein 1 (Fragment) |  | 0.614 |
| orange1.1g043064m|PACid:18124108 | gi|225431497 | RNA-binding protein 8A |  | 0.602 |
| orange1.1g038916m|PACid:18113498 | gi|359386140 | Structural maintenance of chromosomes domain-containing protein |  | 0.595 |
| orange1.1g000919m|PACid:18095071 | gi|357512545 | THO complex subunit |  | 0.566 |
| orange1.1g032043m|PACid:18101981 | gi|359386144 | HAP3-like protein |  | 0.576 |
| orange1.1g026648m|PACid:18105131 | gi|255549562 | Nucleic acid binding protein, putative |  | 0.585 |
| orange1.1g025983m|PACid:18139690 | gi|21363045 | Transcription initiation factor IIB-2 |  | 0.591 |
| orange1.1g003900m|PACid:18096381 | gi|356539190 | Pre-mRNA-splicing factor CWC21-like |  | 0.612 |
| orange1.1g029991m|PACid:18132211 | gi|357474487 | Ribonuclease |  | 1.664 |
|  |  |  |  |  |
| ***Protein metabolism*** |  |  |  |  |
| orange1.1g015333m|PACid:18127488 | gi|241915348 | Ribosomal protein | 0.659 |  |
| **orange1.1g033270m|PACid:18097359** | **gi|46397044** | **60S ribosomal protein L35** | **0.409** | **0.424** |
| **orange1.1g032182m|PACid:18124616** | **gi|255568595** | **60S ribosomal protein L26, putative** | **0.658** | **0.634** |
| **orange1.1g027696m|PACid:18128615** | **gi|441477273** | **60S ribosomal protein L10-3** | **0.536** | **0.432** |
| orange1.1g034868m|PACid:18119092 | gi|448872696 | 60S acidic ribosomal protein P2B | 0.566 |  |
| **orange1.1g025016m|PACid:18117278** | **gi|548774** | **60S ribosomal protein L7a** | **0.470** | **0.498** |
| **orange1.1g033549m|PACid:18127017** | **gi|357443983** | **60S ribosomal protein L36** | **0.663** | **0.321** |
| orange1.1g033702m|PACid:18101071 | gi|42565379 | 60s acidic ribosomal protein | 0.625 |  |
| orange1.1g031766m|PACid:18095314 | gi|73914091 | 60S ribosomal protein L23a-1 | 0.666 |  |
| orange1.1g022424m|PACid:18134235 | gi|449511410 | 60S ribosomal protein L5-like | 0.584 |  |
| orange1.1g033944m|PACid:18127229 | gi|255566866 | 60S acidic ribosomal protein P1, putative | 0.615 |  |
| orange1.1g028425m|PACid:18136127 | gi|148466436 | 60S ribosomal protein L19, partial | 0.539 |  |
| orange1.1g033375m|PACid:18104941 | gi|255546227 | 60S ribosomal protein L31, putative | 0.446 |  |
| **orange1.1g033850m|PACid:18136391** | **gi|357443983** | **60S ribosomal protein L36** | **0.661** | **0.354** |
| orange1.1g037330m|PACid:18099919 | gi|109940162 | 40S ribosomal protein S8 | 0.486 |  |
| **orange1.1g030945m|PACid:18097381** | **gi|380293288** | **60S ribosomal protein L23a, partial** | **0.597** | **0.570** |
| orange1.1g031653m|PACid:18108842 | gi|27923848 | 40S ribosomal protein S15 | 0.442 |  |
| orange1.1g025591m|PACid:18134260 | gi|255569736 | 40S ribosomal protein S6, putative | 0.540 |  |
| orange1.1g031659m|PACid:18093291 | gi|27923849 | 40S ribosomal protein S15 | 0.437 |  |
| orange1.1g023289m|PACid:18131012 | gi|449465358 | 40S ribosomal protein S4-like | 0.660 |  |
| orange1.1g031480m|PACid:18113271 | gi|255571087 | 40S ribosomal protein S11, putative | 0.572 |  |
| **orange1.1g033482m|PACid:18127830** | **gi|225455513** | **40S ribosomal protein S20-2 isoform 1** | **0.655** | **0.665** |
| orange1.1g045444m|PACid:18100699 | gi|148807168 | Putative 40S ribosomal protein S8 | 0.572 |  |
| **orange1.1g042287m|PACid:18104927** | **gi|22096379** | **40S ribosomal protein S30** | **0.591** | **0.663** |
| orange1.1g033931m|PACid:18111091 | gi|470106618 | 40S ribosomal protein S25-4-like | 0.363 |  |
| orange1.1g031854m|PACid:18137306 | gi|82621170 | Cytoplasmic ribosomal protein S13-like | 0.568 |  |
| orange1.1g030800m|PACid:18101357 | gi|225452150 | Ribosome-recycling factor | 0.658 |  |
| orange1.1g038172m|PACid:18091957 | gi|190895944 | Casein kinase II regulatory subunit | 0.479 |  |
| orange1.1g019971m|PACid:18096631 | gi|83305639 | Ribosome biogenesis regulatory protein homolog | 0.459 |  |
| orange1.1g000570m|PACid:18092767 | gi|356576051 | Eukaryotic translation initiation factor 5B-like | 0.509 |  |
| orange1.1g011282m|PACid:18127790 | gi|75171433 | Peptidyl-prolyl cis-trans isomerase FKBP15-3 | 0.283 |  |
| orange1.1g024818m|PACid:18102758 | gi|147856146 | Peptidyl-prolyl cis-trans isomerase | 1.520 |  |
| orange1.1g015812m|PACid:18127720 | gi|75099392 | Subtilisin-like protease | 1.513 |  |
| **orange1.1g037455m|PACid:18100140** | **gi|75099392** | **Subtilisin-like protease** | **5.393** | **2.424** |
| **orange1.1g004265m|PACid:18091291** | **gi|470138103** | **Subtilisin-like protease-like** | **2.643** | **2.354** |
| orange1.1g039166m|PACid:18120398 | gi|257222598 | Subtilisin-like serine protease | 1.731 |  |
| **orange1.1g011108m|PACid:18110416** | **gi|225458529** | **Serine carboxypeptidase II-3-like** | **2.852** | **1.581** |
| orange1.1g012205m|PACid:18107581 | gi|255538024 | Serine carboxypeptidase, putative | 1.718 |  |
| **orange1.1g005260m|PACid:18116644** | **gi|332278204** | **Probable glutamate carboxypeptidase 2** | **2.509** | **1.663** |
| orange1.1g017548m|PACid:18092667 | gi|18419649 | Putative cysteine proteinase | 1.976 |  |
| orange1.1g024783m|PACid:18091771 | gi|42563538 | Cysteine protease-like protein | 2.087 |  |
| orange1.1g012960m|PACid:18091567 | gi|416767 | Cysteine proteinase 4 (Fragment) | 1.937 |  |
| orange1.1g010230m|PACid:18100038 | gi|33347413 | Aspartic protease | 1.577 |  |
| orange1.1g010486m|PACid:18110276 | gi|163256765 | Aspartic protease precursor | 4.024 |  |
| orange1.1g004756m|PACid:18092410 | gi|353441090 | C-terminal domain-containing protein | 1.579 |  |
| orange1.1g038219m|PACid:18106021 | gi|14549156 | Basic 7S globulin | 0.395 |  |
| orange1.1g033055m|PACid:18116367 | gi|26224744 | Type I proteinase inhibitor-like protein | 1.593 |  |
| orange1.1g031804m|PACid:18118759 | gi|464980 | Ubiquitin-conjugating enzyme E2 2 | 0.615 |  |
| **orange1.1g031706m|PACid:18131290** | **gi|18803** | **Polyubiquitin protein** | **0.560** | **0.483** |
| orange1.1g031613m|PACid:18104384 | gi|302595860 | Ubiquitin-60S ribosomal protein L40-1 | 0.633 |  |
| orange1.1g031739m|PACid:18127441 | gi|75332036 | Ubiquitin-conjugating enzyme E2 35 | 0.658 |  |
| orange1.1g032038m|PACid:18126837 | gi|449456369 | Ubiquitin-conjugating enzyme E2 10-like isoform 2 | 0.528 |  |
| orange1.1g029412m|PACid:18093747 | gi|255540105 | Ubiquitin-conjugating enzyme E2-25kD, putative | 0.664 |  |
| orange1.1g047180m|PACid:18127479 | gi|452090852 | Aspartate transaminase, partial | 0.644 |  |
| orange1.1g015072m|PACid:18130718 | gi|20139956 | Probable sarcosine oxidase | 1.845 |  |
| orange1.1g047572m|PACid:18100161 | gi|77627624 | Nitrite reductase | 1.571 |  |
| orange1.1g028623m|PACid:18135510 | gi|17865558 | 60S ribosomal protein L13a-4 |  | 0.634 |
| orange1.1g028624m|PACid:18107114 | gi|730450 | 60S ribosomal protein L13-2 |  | 0.511 |
| orange1.1g028729m|PACid:18109737 | gi|20143885 | 60S ribosomal protein L19-1 |  | 0.286 |
| orange1.1g032760m|PACid:18133304 | gi|449449533| | 40S ribosomal protein S24-2-like |  | 0.482 |
| orange1.1g045953m|PACid:18126147 | gi|359484922 | Probable prefoldin subunit 1 |  | 0.654 |
| orange1.1g031977m|PACid:18129366 | gi|255559796 | Prefoldin subunit, putative |  | 0.658 |
| orange1.1g039265m|PACid:18120392 | gi| 75099062 | Subtilisin-like protease SDD1 |  | 2.353 |
| orange1.1g036132m|PACid:18105794 | gi|14549156 | Basic 7S globulin |  | 0.558 |
| orange1.1g037784m|PACid:18108812 | gi|14549156 | Basic 7S globulin |  | 0.376 |
| orange1.1g034261m|PACid:18122821 | gi|289524946 | Putative cystein proteinase inhibitor |  | 0.609 |
| orange1.1g031498m|PACid:18099476 | gi|227184175 | SKP1-like protein |  | 0.666 |
| orange1.1g000012m|PACid:18114095 | gi|172045749 | E3 ubiquitin-protein ligase UPL1 |  | 0.637 |
| orange1.1g034043m|PACid:18131846 | gi|55859483 | Ubiquitin-like protein SMT3 |  | 0.625 |
| orange1.1g024960m|PACid:18126465 | gi|75243459 | Ubiquitin carboxyl-terminal hydrolase 13 |  | 0.584 |
| orange1.1g016039m|PACid:18127583 | gi|225435357 | Phenylalanine ammonia-lyase |  | 0.520 |
| orange1.1g010897m|PACid:18135444 | gi|95116497 | Putative glutamate decarboxylase |  | 1.895 |
| orange1.1g035637m|PACid:18113682 | gi|470125394 | Primary amine oxidase-like |  | 1.591 |
|  |  |  |  |  |
| ***Cell transport*** |  |  |  |  |
| orange1.1g022337m|PACid:18105404 | gi|75333593 | ABC transporter I family member 17 | 4.831 |  |
| orange1.1g022966m|PACid:18097320 | gi|82659447 | Aquaporin | 1.897 |  |
| orange1.1g023854m|PACid:18101790 | gi|224102711 | Porin/voltage-dependent anion-selective channel protein | 1.854 |  |
| **orange1.1g034153m|PACid:18097165** | **gi|585960** | **Protein transport protein Sec61 subunit β** | **2.996** | **0.596** |
| orange1.1g009582m|PACid:18132198 | gi|21450871 | Putative permease 1 (Nucleobase-ascorbate transporter 6, NAT6) | 1.628 |  |
| orange1.1g021703m|PACid:18138244 | gi|11177028 | Ferritin 1, partial | 1.501 |  |
| orange1.1g032005m|PACid:18111723 | gi|470110517 | Putative phosphatidylglycerol/phosphatidylinositol transfer protein DDB_G0282179-like | 1.604 |  |
| orange1.1g023623m|PACid:18116182 | gi|28380129 | Syntaxin-132 | 0.655 |  |
| orange1.1g047939m|PACid:18110974 | gi|289584365 | Syntaxin-like protein | 0.658 |  |
| orange1.1g031983m|PACid:18138726 | gi|225463791 | Mitochondrial intermembrane space import and assembly protein 40 | 0.652 |  |
| orange1.1g002208m|PACid:18105400 | gi|31580853 | Plasma membrane H^+^-ATPase |  | 1.524 |
| orange1.1g016996m|PACid:18103473 | gi|3334117 | ADP,ATP carrier protein 2, mitochondrial |  | 1.556 |
| orange1.1g026478m|PACid:18137084 | gi|470142506 | Vesicle-associated protein 1-3-like |  | 1.506 |
| orange1.1g034237m|PACid:18131173 | gi|14423789 | Non-specific lipid-transfer protein 2 |  | 0.419 |
| orange1.1g031445m|PACid:18093361 | gi|75161488 | Non-specific lipid-transfer protein-like protein At5g64080 |  | 0.646 |
|  |  |  |  |  |
| ***Biological regulation and signal transduction*** | |  |  |  |
| orange1.1g002255m|PACid:18095138 | gi|1174718 | Probable receptor protein kinase TMK1 | 1.573 |  |
| orange1.1g007200m|PACid:18094921 | gi|388325711 | Chain A, structural basis for the impact of phosphorylation on plant receptor- like kinase bak1 activation | 1.774 |  |
| orange1.1g006982m|PACid:18119635 | gi|255572716 | ATP binding protein, putative | 1.525 |  |
| orange1.1g022973m|PACid:18095810 | gi|255570037 | DUF26 domain-containing protein 1 precursor, putative | 1.710 |  |
| **orange1.1g026017m|PACid:18095038** | **gi|75248508** | **Purple acid phosphatase 8** | **2.823** | **1.571** |
| orange1.1g027915m|PACid:18138796 | gi|62319782 | Endomembrane-associated protein (Plasma membrane-associated cation-binding protein 1) | 2.192 |  |
| **orange1.1g031226m|PACid:18133985** | **gi|289064983** | **Calmodulin 24-like protein** | **0.445** | **0.574** |
| **orange1.1g009980m|PACid:18139211** | **gi|85540592** | **DMI3 protein** | **0.611** | **0.665** |
| orange1.1g037001m|PACid:18103372 | gi|6016428 | Putative casein kinase II subunit beta-4 |  | 0.614 |
| orange1.1g011819m|PACid:18104748 | gi|52077492 | Putative protein kinase ADK1 |  | 0.594 |
| orange1.1g008055m|PACid:18115829 | gi|33772201 | Protein kinase |  | 1.665 |
| orange1.1g011349m|PACid:18111516 | gi|255558866 | Receptor protein kinase, putative |  | 1.505 |
| orange1.1g021044m|PACid:18121448 | gi|470120914 | Putative G3BP-like protein-like |  | 0.410 |
|  |  |  |  |  |
| ***Cell wall and cytoskeleton metabolism*** |  |  |  |  |
| orange1.1g032890m|PACid:18116884 | gi|14423860 | Profilin-3 | 0.608 |  |
| orange1.1g013335m|PACid:18103942 | gi|135444 | Tubulin beta-1 chain (Fragment) | 0.567 |  |
| orange1.1g000146m|PACid:18138585 | gi|75250086 | Protein MOR1 (Protein MICROTUBULE ORGANIZATION 1) | 0.622 |  |
| orange1.1g013977m|PACid:18131749 | gi|241865168 | Putative microfibril-associated protein | 0.658 |  |
| orange1.1g009911m|PACid:18119842 | gi|195976596 | Katanin p60 | 0.633 |  |
| orange1.1g035767m|PACid:18137424 | gi|449454512 | Fasciclin-like arabinogalactan protein 8-like | 0.585 |  |
| orange1.1g013841m|PACid:18098238 | gi|224129194 | Fasciclin-like arabinogalactan protein | 0.644 |  |
| orange1.1g038806m|PACid:18110934 | gi|297600120 | Os02g0816500 protein (Putative tubulin folding cofactor A) |  | 0.638 |
| orange1.1g013724m|PACid:18116745 | gi|59799374 | Dynamin-2B |  | 0.608 |
|  |  |  |  |  |
| ***JA biosynthesis*** |  |  |  |  |
| orange1.1g002649m|PACid:18100582 | gi|147845411 | Lipoxygenase | 5.427 |  |
| orange1.1g002706m|PACid:18100674 | gi|147845411 | Lipoxygenase | 2.162 |  |
| orange1.1g007504m|PACid:18094360 | gi|62286589 | Peroxisomal acyl-coenzyme A oxidase 1 (Long-chain acyl-CoA oxidase) | 1.501 |  |
| orange1.1g015862m|PACid:18094276 | gi|225460240 | 12-oxophytodienoate reductase 2 | 1.853 |  |
|  |  |  |  |  |
| ***Others*** |  |  |  |  |
| orange1.1g020406m|PACid:18094781 | gi|225436091 | Probable carboxylesterase 15 | 1.632 |  |
| orange1.1g033119m|PACid:18119666 | gi|157072867 | Acyl-carrier-protein | 0.661 |  |
| orange1.1g018681m|PACid:18119341 | gi|224128073 | Eugenol O-methyltransferase family protein | 1.726 |  |
| orange1.1g020245m|PACid:18127660 | gi|75249391 | Nitrile-specifier protein 5 | 6.507 |  |
| orange1.1g017213m|PACid:18112020 | gi|15214076 | Putative MO25-like protein At5g47540 | 1.747 |  |
| orange1.1g043114m|PACid:18107934 | gi|75911513 | SP-4 | 1.595 |  |
| orange1.1g038676m|PACid:18119881 | gi|225442747 | Gibberellin 20 oxidase 3 | 1.870 |  |
| orange1.1g030936m|PACid:18112231 | gi|149392202 | Heat-and acid-stable phosphoprotein (Fragment) | 0.628 |  |
| orange1.1g015780m|PACid:18126402 | gi|297609742 | Os09g0500900 | 1.709 |  |
| orange1.1g004037m|PACid:18110620 | gi|332278165 | C2 domain-containing protein At1g53590 | 0.563 |  |
| orange1.1g033277m|PACid:18133585 | gi|122206714 | Protein SPIRAL1-like 2 | 0.562 |  |
| **orange1.1g033992m|PACid:18127849** | **gi|388516273** | **Unknown** | **0.582** | **0.601** |
| orange1.1g026104m|PACid:18104053 | gi|224137716 | Predicted protein | 0.648 |  |
| **orange1.1g007522m|PACid:18117358** | **gi|224119738** | **Predicted protein** | **0.429** | **0.558** |
| orange1.1g023009m|PACid:18099053 | gi|462397932 | Hypothetical protein PRUPE_ppa009475mg | 1.504 |  |
| orange1.1g044796m|PACid:18104188 | gi|125532432 | Hypothetical protein OsI_34106 | 0.648 |  |
| orange1.1g024023m|PACid:18136528 | gi|224130792 | Predicted protein | 1.531 |  |
| orange1.1g004971m|PACid:18108089 | gi|224056419 | Predicted protein | 2.653 |  |
| orange1.1g025944m|PACid:18127077 | gi|224126367 | Predicted protein | 1.643 |  |
| orange1.1g046297m|PACid:18115281 | gi|223529439 | Putative uncharacterized protein | 1.687 |  |
| **orange1.1g021530m|PACid:18114238** | **gi|224116144** | **Predicted protein** | **1.933** | **1.844** |
| orange1.1g046091m|PACid:18123894 | gi|297738676 | Unnamed protein product | 1.536 |  |
| orange1.1g013474m|PACid:18139596 | gi|302143430 | Unnamed protein product | 0.622 |  |
| orange1.1g013758m|PACid:18129550 | gi|297744813 | Unnamed protein product, partial | 0.597 |  |
| orange1.1g033117m|PACid:18096495 | gi|462418649 | Hypothetical protein PRUPE_ppb014410mg, partial | 0.577 |  |
| orange1.1g007639m|PACid:18107642 | gi|449455244 | Uncharacterized protein LOC101217045 | 0.645 |  |
| orange1.1g028276m|PACid:18112198 | gi|116791668 | Unknown | 0.615 |  |
| orange1.1g015055m|PACid:18108853 | gi|356572648 | Uncharacterized protein LOC100818037 | 0.663 |  |
| orange1.1g038463m|PACid:18099611 | - | - | 0.600 |  |
| orange1.1g013756m|PACid:18106420 | gi|297741224 | Unnamed protein product | 0.627 |  |
| orange1.1g006196m|PACid:18128993 | gi|297831220 | Hypothetical protein ARALYDRAFT_479925 | 0.546 |  |
| orange1.1g021202m|PACid:18097011 | gi|115434880 | Os01g0179300 | 0.352 |  |
| orange1.1g031113m|PACid:18130280 | gi|462404702 | Hpothetical protein PRUPE_ppb020505mg, partial | 0.635 |  |
| **orange1.1g018078m|PACid:18132803** | **gi|302773283** | **Hpothetical protein SELMODRAFT_440984** | **0.609** | **0.640** |
| **orange1.1g046007m|PACid:18124742** | **gi|297745702** | **Unnamed protein product** | **0.636** | **0.145** |
| orange1.1g010944m|PACid:18114522 | gi|296086984 | Unnamed protein product | 0.660 |  |
| orange1.1g002540m|PACid:18106747 | gi|225437402 | Uncharacterized protein C57A7.06 | 0.566 |  |
| orange1.1g041481m|PACid:18117567 | gi|356518040 | Uncharacterized protein LOC100815978 | 0.637 |  |
| orange1.1g045441m|PACid:18114079 | gi|356527146 | Uncharacterized protein LOC100812080 | 0.449 |  |
| orange1.1g020841m|PACid:18109560 | gi|224108832 | Predicted protein | 0.636 |  |
| orange1.1g010141m|PACid:18116134 | gi|194691976 | Unknown | 0.529 |  |
| orange1.1g022179m|PACid:18128288 | gi|296084798 | Unnamed protein product | 0.584 |  |
| orange1.1g029705m|PACid:18128557 | gi|356569145 | LOW QUALITY PROTEIN: uncharacterized GPI-anchored protein At1g27950-like | 0.530 |  |
| orange1.1g011168m|PACid:18106831 | gi|224166900 | Predicted protein | 0.628 |  |
| orange1.1g028159m|PACid:18131993 | gi|462395885 | Hypothetical protein PRUPE_ppa011364mg | 1.772 |  |
| orange1.1g014901m|PACid:18121745 | gi|388507866 | Unknown | 1.776 |  |
| orange1.1g031460m|PACid:18128345 | gi|357463399 | Hypothetical protein MTR_3g087510 | 1.640 |  |
| **orange1.1g034789m|PACid:18110744** | **gi|296086561** | **Unnamed protein product** | **2.455** | **0.557** |
| orange1.1g020314m|PACid:18131725 | gi|297742883 | Unnamed protein product | 1.847 |  |
| orange1.1g004921m|PACid:18091994 | gi|308081381 | Uncharacterized protein LOC100502422 | 0.637 |  |
| orange1.1g004038m|PACid:18110587 | gi|550342961 | Uncharacterized protein | 3.308 |  |
| orange1.1g032651m|PACid:18135561 | gi|195626548 | Hypothetical protein | 0.569 |  |
| orange1.1g019238m|PACid:18121085 | gi|224153140 | Predicted protein | 1.535 |  |
| **orange1.1g028549m|PACid:18101102** | **gi|15237822** | **Remorin family protein** | **2.206** | **0.440** |
| orange1.1g046829m|PACid:18093894 | gi|326501042 | Predicted protein | 1.546 |  |
| **orange1.1g022834m|PACid:18134215** | **gi|413937314** | **Hypothetical protein ZEAMMB73_741597** | **1.522** | **1.792** |
| orange1.1g009300m|PACid:18134158 | gi|147772590 | Hypothetical protein VITISV_008972 | 0.594 |  |
| orange1.1g027031m|PACid:18114002 | gi|147819084 | Hypothetical protein VITISV_016707 | 0.521 |  |
| orange1.1g016526m|PACid:18115913 | gi|225425084 | Uncharacterized protein LOC100249627 | 0.554 |  |
| **orange1.1g041939m|PACid:18094259** | **gi|462411292** | **Hypothetical protein PRUPE_ppa013243mg** | **0.460** | **0.424** |
| orange1.1g011975m|PACid:18123199 | gi|225439119 | Uncharacterized protein | 0.403 |  |
| orange1.1g003124m|PACid:18111441 | gi|224059448 | Predicted protein | 0.430 |  |
| orange1.1g015276m|PACid:18127759 | gi|147783651 | Hypothetical protein VITISV_039964 | 0.550 |  |
| orange1.1g017337m|PACid:18113073 | gi|388492624 | Uncharacterized protein | 0.538 |  |
| orange1.1g045218m|PACid:18132696 | gi|224071796 | Predicted protein | 0.649 |  |
| orange1.1g004252m|PACid:18121959 | gi|258644606 | Hypothetical protein | 0.649 |  |
| **orange1.1g017245m|PACid:18100940** | **gi|363808216** | **Uncharacterized protein LOC100798871** | **2.034** | **1.582** |
| orange1.1g041485m|PACid:18111432 | gi|462401687 | Hypothetical protein PRUPE_ppa012585mg | 2.787 |  |
| orange1.1g018445m|PACid:18128806 | gi|255544768 | Oxidoreductase, putative |  | 1.749 |
| orange1.1g016941m|PACid:18115382 | gi|34395735 | Early nodulin-like protein 2 |  | 0.499 |
| orange1.1g032632m|PACid:18110899 | gi|449432287 | Uncharacterized protein LOC101218027 |  | 0.660 |
| orange1.1g025984m|PACid:18125303 | gi|224096724 | Predicted protein |  | 0.640 |
| orange1.1g026270m|PACid:18106041 | gi|449515382 | Uncharacterized LOC101209829, partial |  | 1.510 |
| orange1.1g011105m|PACid:18118026 | gi|296084906 | Unnamed protein product |  | 0.522 |
| orange1.1g000790m|PACid:18101369 | gi|224112303 | Predicted protein |  | 0.641 |
| orange1.1g019927m|PACid:18115080 | gi|24461852 | Hypothetical protein |  | 0.583 |
| orange1.1g031435m|PACid:18103914 | gi|462401663 | Hypothetical protein PRUPE_ppa012435mg |  | 0.341 |
| orange1.1g005888m|PACid:18092262 | gi|224066211 | Predicted protein |  | 0.474 |
| orange1.1g047864m|PACid:18122291 | gi|470120729 | Uncharacterized protein LOC101304270 |  | 0.602 |
| orange1.1g001084m|PACid:18113450 | gi|224100645 | Predicted protein |  | 0.648 |
| orange1.1g025643m|PACid:18110039 | gi|225435822 | Uncharacterized protein LOC100250144 |  | 0.643 |
| orange1.1g035807m|PACid:18101568 | gi|224072600 | Predicted protein |  | 0.550 |
| orange1.1g025469m|PACid:18134510 | gi|224135809 | Predicted protein |  | 0.420 |
| orange1.1g046011m|PACid:18133009 | gi|326510561 | Predicted protein |  | 0.564 |
| orange1.1g010497m|PACid:18099568 | gi|356544429 | Uncharacterized protein LOC100812553, partial |  | 0.614 |
| orange1.1g010864m|PACid:18119052 | gi|224075226 | Predicted protein |  | 0.556 |
| orange1.1g028835m|PACid:18102498 | gi|460386852 | Uncharacterized protein LOC101260817 isoform 1 |  | 0.382 |
| orange1.1g045761m|PACid:18099576 | gi|297744467 | Unnamed protein product |  | 0.628 |
| orange1.1g032418m|PACid:18107958 | gi|351734466 | Uncharacterized protein LOC100306069 |  | 0.593 |
| orange1.1g014354m|PACid:18109759 | gi|413955910 | Hypothetical protein ZEAMMB73_763228 |  | 0.660 |
| orange1.1g008110m|PACid:18115170 | gi|224065188 | Predicted protein |  | 0.666 |
| orange1.1g043780m|PACid:18092908 | gi|462404971 | Hypothetical protein PRUPE_ppa007016mg |  | 1.550 |
| orange1.1g019052m|PACid:18094310 | gi|297739546 | Unnamed protein product |  | 0.594 |
| orange1.1g004329m|PACid:18127311 | gi|462397202 | Hypothetical protein PRUPE_ppa001837mg |  | 1.522 |
| orange1.1g008840m|PACid:18116996 | gi|224111534 | Predicted protein |  | 2.073 |
| orange1.1g034185m|PACid:18136524 | gi|255551743 | Conserved hypothetical protein |  | 0.587 |
| orange1.1g010119m|PACid:18091366 | gi|224063515 | Predicted protein |  | 0.655 |
| orange1.1g021055m|PACid:18135423 | gi|462411218 | Hypothetical protein PRUPE_ppa012346mg |  | 0.620 |
| orange1.1g009565m|PACid:18107528 | gi|388517133 | Uncharacterized protein |  | 0.621 |
| orange1.1g019324m|PACid:18131127 | gi|224124078 | Predicted protein |  | 0.660 |
| orange1.1g045361m|PACid:18130320 | gi|218188793 | Hypothetical protein OsI_03150 |  | 0.623 |

Control and Al-toxic samples for CS (CG) were labeled with 119 and 118 (117 and 115) tags, respectively.
