# Supplementary material for: Root iTRAQ protein profile analysis of two Citrus species differing in aluminum-tolerance in response to long-term aluminum-toxicity
Source: BMC Genomics. 2015 Nov 16;16:949. doi: 10.1186/s12864-015-2133-9 (PMC4647617; doi:10.1186/s12864-015-2133-9)
Supplement: Additional file 3: — Specific primer pairs used for qRT-PCR expression analysis. (DOC 40 kb) [file 12864_2015_2133_MOESM3_ESM.doc]

**Additional file 3: Table S1.** Specific primer pairs used for qRT-PCR expression analysis

| Accession | Homology | Gene | Forward primer (5´→3´) | Reverse primer (5´→3´) |
| --- | --- | --- | --- | --- |
| orange1.1g012433m|PACid:18106261 | gi|281426908 | *ATP sulfurylase 1* | GATCGTTTCCGATTGTCACCTGC | ACATCATCTGCCTTTGTATAGCCTCCT |
| orange1.1g020528m|PACid:18133307 | gi|34099833 | *O-acetylserine (thiol)lyase, partial* | TTTGAAAACCCAGCAAACCCAAAG | TCCAGGGATGAAGCCAGCACC |
| orange1.1g042301m|PACid:18120525 | gi|380863042 | *Glutathione transferase, partial* | AAGAGCGGCTTATTGTTTGCTGAAG | TCACTGACTTTGCGGAAATAGACGAG |
| orange1.1g030845m|PACid:18093214 | gi|75154467 | *Probable glutathione peroxidase 4* | 5GTCAGGAGGCTCACGAATTTGCA | CCATCGGAGAAGTGGTTGGGCTAT |
| orange1.1g042356m|PACid:18092056 | gi|378724814 | *Catalase* | ACGCCTGTCATTGTCCGCTTCT | TCTTGGATGTGAGACTTTGGGTTAGGT |
| orange1.1g018811m|PACid:18128473 | gi|110007377 | *Peroxidase* | TCAAGAGCTGTTTTCGACACCCG | GCTGACCAAACCGCCATCTGA |
| orange1.1g040384m|PACid:18119464 | gi|1171937 | *Oxalate oxidase 2* | GCTGATCCCGATCCTCTACAAGACA | TTCCACCAACAGCAAGGTCAACTC |
| orange1.1g042021m|PACid:18116199 | gi|21264375 | *Blue copper protein* | ATGACTCGGGATGGACAGTAGGGTT | CTGAGGCAGCACAGTAATGACAAGC |
| orange1.1g026030m|PACid:18104367 | gi|301341860 | *Glyoxylase I, partial* | CGCTAACAATCCGGGTCTTCACA | CGGCTTCCCAAAAGTCCAAACA |
| orange1.1g027699m|PACid:18095456 | gi|3913733 | *Hydroxyacylglutathione hydrolase* | TGTCAATCTCACCACCGTTCTCACTAC | TGGGTCTTCTCCCTCTTTTCCAGTC |
|  | JN580571 | *β-Tubulin* | CCCTTTACGACATCTGTTTCCG | TGGCATCCCACATTTGCTG |
